# Comparing a Virtual Reality–Based Simulation App (VR-MRI) With a Standard Preparatory Manual and Child Life Program for Improving Success and Reducing Anxiety During Pediatric Medical Imaging: Randomized Clinical Trial

**DOI:** 10.2196/22942

**Published:** 2021-09-22

**Authors:** Chelsea Stunden, Kirsten Stratton, Sima Zakani, John Jacob

**Affiliations:** 1 Department of Pediatrics Faculty of Medicine University of British Columbia Vancouver, BC Canada; 2 Digital Lab at BC Children's Hospital Vancouver, BC Canada; 3 Department of Child Life BC Children's Hospital Vancouver, BC Canada; 4 City University of London London United Kingdom

**Keywords:** virtual reality, magnetic resonance imaging simulation, certified child life specialists, pediatrics, magnetic resonance imaging procedures, alternatives to sedation, preparing children for MRI

## Abstract

**Background:**

The experience of undergoing magnetic resonance imaging (MRI) can be anxiety provoking, particularly for pediatric patients and their families. Alternative methods to improve success and experiences without the use of sedation are needed.

**Objective:**

This study aims to compare the effectiveness of a virtual reality (VR)—based simulation app (VR-MRI) with a standard preparatory manual (SPM) and a hospital-based Child Life Program (CLP) on success and anxiety during a simulated pediatric MRI scan. Our secondary aim is to compare caregivers’ reported anxiety, procedural data, caregiver usability, child satisfaction, and fun.

**Methods:**

This unblinded, randomized, triple-arm clinical trial involved 92 children aged 4-13 years and their caregivers. Recruitment was conducted through posters, public libraries, community centers, and social media. At a 2-hour session, participants were instructed to prepare for a simulated MRI head scan using one of three randomly assigned preparation materials: the VR-MRI app, SPM, or the CLP. Data were collected before preparation, during a simulated MRI head scan, and after the simulated scan. The primary outcomes were the success of the simulated MRI scan (MoTrak head motion tracking system), and child-reported anxiety (Venham picture test). We secondarily measured caregivers’ reported anxiety (short State-Trait Anxiety Inventory), procedural data (minutes), usability (Usefulness, Satisfaction, and Ease of Use Questionnaire), and child-reported satisfaction and fun (visual analog scales).

**Results:**

A total of 84 participants were included in the final analysis (VR-MRI: 30/84, 36%; SPM: 24/84, 29%; and CLP: 30/84, 36%). There were no *clinically* significant differences between the groups in terms of success during the MRI simulation (*P=*.27) or the children’s reported anxiety at any timepoint (timepoint 1, *P*=.99; timepoint 2, *P*=.008; timepoint 3, *P*=.10). Caregivers reported being significantly more anxious after preparing with the manual than caregivers in the other 2 groups (*P*<.001). Child and caregiver anxiety had a significant relationship, increasing together with moderate effect (r_84_=0.421; *P*<.001). Participants using VR-MRI took the most time to prepare (*P*<.001) and participants using the manual took the least time (*P*<.001). No statistically significant relationships were found between time preparing and time completing the simulated assessment (*P=*.13). There were no differences found in ease of use (*P*=.99), ease of learning (*P*=.48), and usefulness (*P*=.11) between the groups; however, caregivers reported being significantly more satisfied with the VR-MRI app and CLP than SPM (*P*<.001). Children reported the most satisfaction with the CLP (*P*<.001). There were no differences in how much fun the preparation materials were perceived to be (*P*=.37).

**Conclusions:**

Digital preparation experiences using VR-based media could be a viable solution to improve the success of nonsedated MRI scans, with outcomes comparable with hospital-based in-person preparatory programs. Future research should focus on validating the results in a real MRI setting.

**Trial Registration:**

Clinicaltrials.gov NCT03931382; https://clinicaltrials.gov/ct2/show/NCT03931382

## Introduction

### Background

Previous reports estimate that between 50% and 75% of pediatric patients experience elevated anxiety and distress before a new medical procedure, such as medical imaging [1,2]. Poor management of this anxiety has an impact on patient experience by causing undue psychological and physiological distress [1-4] and movement during the procedure, which can significantly reduce the diagnostic quality of medical imaging [5]. It also has a ripple effect by causing additional logistical, operational, and economic burdens on the health care system and patients [6,7]. Short-term consequences include pain or discomfort, anxiety, crying, and poor cooperation during the procedure [1,8,9]. In the long term, negative experiences can evolve into posttraumatic stress syndrome, fear, changes in pain perception and coping effectiveness, avoidance of medical care, and phobias, causing difficulties during future medical experiences [9,10]. These consequences can lead to additional resource requirements, system impacts, and costs of care that are reported to be 3.24-9.56 times higher for sedated patients than for those who can complete the procedure unsedated [6]. Thus, it is important to properly and effectively assess pediatric patients before medical procedures and improve compliance with appropriate preparation techniques whenever feasible.

At our institution, >4500 pediatric patients undergo magnetic resonance imaging (MRI) each year, with approximately 45% of patients requiring sedation. In the 2017 to 2018 fiscal year, the waitlist for sedated MRI reached a significant point where some patients were forecasted to endure a wait of >1 year for their scheduled appointments. To address this, the hospital launched a coordinated strategy that included increased operational capacity for medical imaging and funding for clinical and support services. Certified child life specialists (CCLSs), trained to prepare patients and families for procedures, were among these resources. CCLSs make targeted efforts to reduce the need for procedural sedation through training and exposure therapy techniques involving simulated procedures and therapeutic play [11-14]. Workshops or programs delivered by CCLSs have been implemented in >400 North American health care settings [13] and are considered a key factor in enabling some patients to undergo imaging without the use of sedation, where it may have otherwise been indicated to address high preprocedural anxiety [11,15].

The Child Life Program (CLP) at our hospital uses a replica of an MRI unit to orientate and practice the process with patients before the true imaging procedure. This method has been documented in the literature by other investigators [15-17]. However, capacity limitations still exist, and there are socioeconomic costs and logistical considerations of only having these units available on-site at tertiary care facilities. To mitigate this, our hospital offers physical materials such as preparatory manuals and telephone and email consultations to orient patients before the procedure. These methods have also been discussed in the literature [12,14,18,19]; however, there is some conflicting evidence regarding their efficacy [20].

Recently, virtual reality (VR), a computer-generated simulation of a 3D environment that can be explored and interacted with by the use of a head-mounted display (HMD), has emerged as an effective solution for reducing anxiety in a variety of pediatric psychological applications [21,22], including preprocedural anxiety [23-32]. Although the use of VR in hospital settings is promising, to our knowledge, only one study has compared it with the CLP regarding anxiety [30]. We also found only one small meta-analysis comparing different types of preparation programs regarding objective image quality [33]. Of the studies that have been conducted to evaluate VR in procedural preparation thus far, most have focused primarily on self-reporting and survey metrics [27,28,30]. There is a need for rigorous randomized clinical trials using innovative methods to establish the safety, feasibility, and efficacy of alternatives to sedation in preparing pediatric patients for medical imaging procedures before applying them in real pediatric clinical settings [21,34-36].

### Objectives

In this context, this study aimed to compare the effectiveness of a custom-developed VR-based intervention with established hospital alternatives in preparing children aged 4-13 years for a simulated medical imaging procedure. We hypothesized that a VR app (VR-MRI) based on experiential learning [37] and social cognitive theory [38] would be effective in preparing pediatric patients for a successful MRI experience, reducing periprocedural anxieties and, thus, noncompliant behaviors contributing to poor image quality or acquisition. We secondarily hypothesize that:

- VR-MRI would reduce caregiver anxiety;
- Children’s anxiety would be related to their caregiver’s anxiety;
- More time practicing would result in periprocedural efficiency;
- Caregivers would be satisfied with VR-MRI, and it would be perceived as useful and easy to learn;
- Children would be satisfied with VR-MRI, and it would be perceived as fun to use.

Ultimately, we suggest that digital preparation apps using immersive media, such as our VR-MRI solution, could improve outcomes and patient experience by introducing standardized, accessible, and independently repeatable opportunities for experiential learning and preprocedural simulated practice. Furthermore, we suggest that interventions such as this could reduce costs and burden of health systems [7] through increased efficiency and reduced need for pharmaceutical intervention or anesthesia services to improve compliance during medical imaging.

## Methods

### VR-MRI Design

The VR-MRI media was custom-designed by the British Columbia Children's Hospital Digital Lab. The development process was informed by the literature [39], encompassing recent design strategy recommendations for VR [34]. The development of VR-MRI included iterative consultation and testing with a multidisciplinary team of approximately 8 stakeholders, including CCLSs, radiology technicians, child psychologists, health system administrators, and the research team. The curriculum focused on the same material included in the standard preparatory manual (SPM) and CLP: developing rapport with medical professionals, getting comfortable with the hospital setting and medical equipment, assessing reactions to pictures of a real MRI, discussing the upcoming medical procedure, and getting comfortable with earplugs, headphones, loud noises, restraints, the head coil, going into and remaining inside the bore, and holding still.

We used the agile development methodology to cycle through user experience design, development, alpha testing, and beta testing [40]. The content in our study was custom developed in Unity (Unity Technologies version 2018.4.9f1) and displayed on a Samsung S9 mobile phone that was used with a MERGE VR headset (Merge Labs Inc). The headset was selected for its balance of quality (eg, repeat use, compatibility with hygiene solutions, and interpupillary adjustments designed for children) and affordability, priced at Can $40 (US $32). The headset requires a mobile phone to be inserted into an embedded front panel that is viewable to the users. We used AirServer Connect (App Dynamic ehf) to mirror the VR-MRI sequence in real time on a tablet device for caregivers to watch in parallel.

### VR-MRI App

#### Tutorial

**Figure 1:**
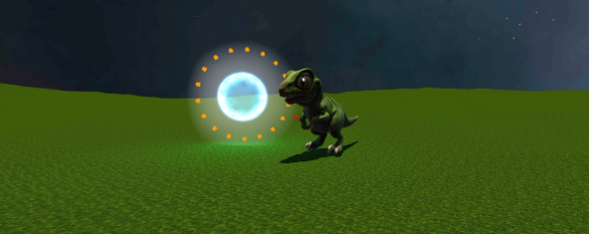
A screenshot of the virtual reality magnetic resonance imaging tutorial.

A tutorial was designed to help the user learn how to interact with the different elements presented in the virtual environment. The tutorial included a dinosaur in outer space that taught the user how to interact with the elements, see in 360°, and interact with hotspots (referred to as teleportation devices). An image of the tutorial is shown in Figure 1.

#### Tour Sequence

**Figure 2:**
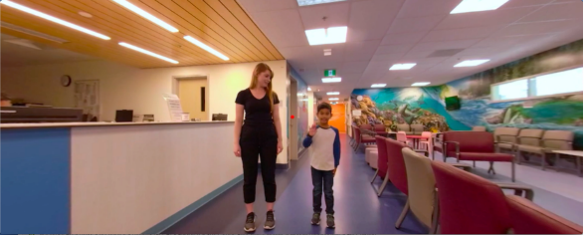
A screenshot of the introduction to hospital staff and a peer in the reception area.

**Figure 3:**
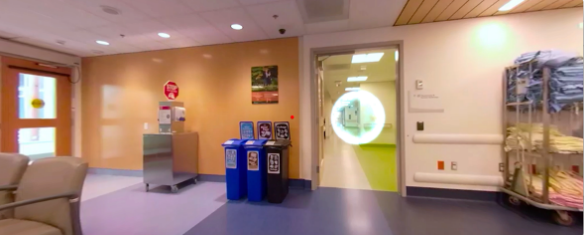
A screenshot of the interactive hotspots used to transition through rooms and sequences.

The tour sequence aimed to get the user comfortable at the hospital, with the medical staff, and with the medical equipment. For creating a sense of safety in the hospital, users were first introduced to a radiologist and a peer in the reception area (Figure 2). They led the user through an interactive guided tour of the hospital reception areas, the imaging room, and the steps of a head scan*.* Each room can be passively explored by rotating the head in 360°. To introduce some autonomy, we also implemented interactive hotspots that needed to be activated by the user to transition between rooms or sequences (Figure 3).

**Figure 4:**
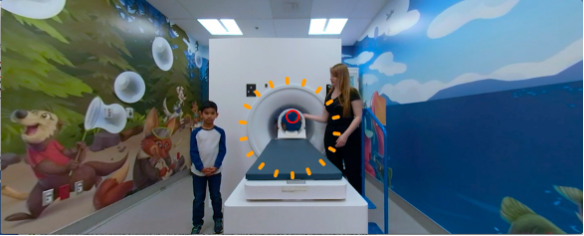
A screenshot of the sequence focusing the user’s attention on the head coil.

**Figure 5:**
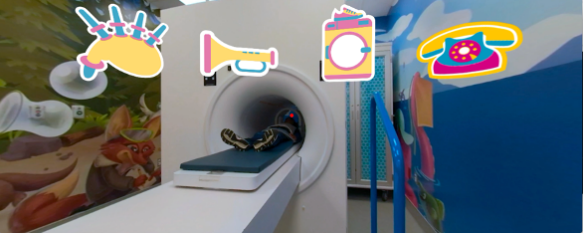
A screenshot of narrative elements used to describe the magnetic resonance imaging sounds.

In the MRI room, the procedural steps of the head scan were introduced. The MRI procedure involved wearing earplugs, headphones, putting on the restraints (referred to as seatbelts), putting on the head coil (referred to as a helmet), listening to sounds (referred to as familiar sounds), and going into the bore (referred to as the tunnel). If the user was distracted during the introduction of important information, cued by not looking in the appropriate direction, the sequence would stall until the user’s attention was refocused appropriately (Figure 4). To improve the child’s ability to cope with the MRI sounds, which are very loud and noisy, we introduced them as familiar sounds using a narrative element while the sounds played. To further build comfort with the loud noises, users were given the opportunity to play with the sounds via buttons (Figure 5).

After the introduction to the procedure, the user was invited to watch a peer successfully complete an MRI scan and then invited to complete one themselves. At this point, the user could choose to either watch the peer again or attempt it themselves by selecting an interactive hotspot.

#### The Virtual MRI Experience and Real-time Feedback Game

**Figure 6:**
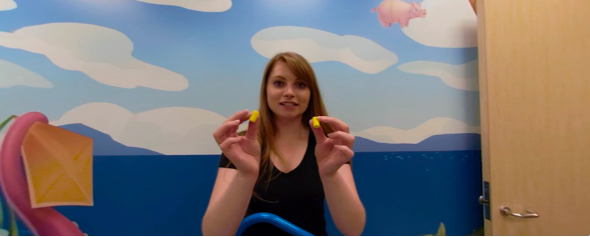
A screenshot of putting in earplugs during the user’s virtual imaging experience.

**Figure 7:**
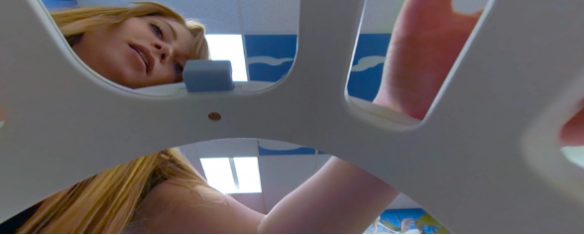
A screenshot of putting on the head coil during the user’s virtual imaging experience.

During the user’s virtual imaging experience (first-person view), the radiologist prepared them for the scan by inserting the earplugs and putting on the headphones (Figure 6). Once the safety equipment was put on, the user was instructed to lie down. For safety purposes, the research assistant helped facilitate this movement on a yoga mat. After the participant laid down, the scene progressed to attaching the head coil (Figure 7). The radiologist then placed them in the tunnel.

**Figure 8:**
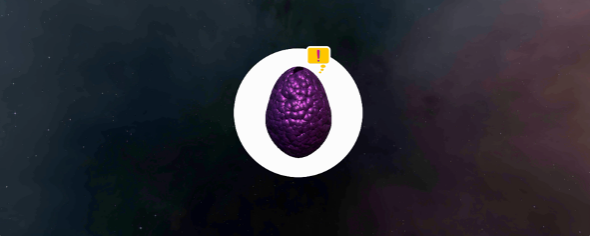
A screenshot of the real-time feedback during movement, measured using the mobile phone gyroscope.

Once in the tunnel, a sequence began to engage the patient in game-based activities to build capacity for self-regulation of their movements during the scan. The user was invited to interact with a dinosaur egg that offered real-time feedback on indicators of movement (such as tilt, shake, rotation, or swing) measured by the mobile phone’s gyroscope (Figure 8).

**Figure 9:**
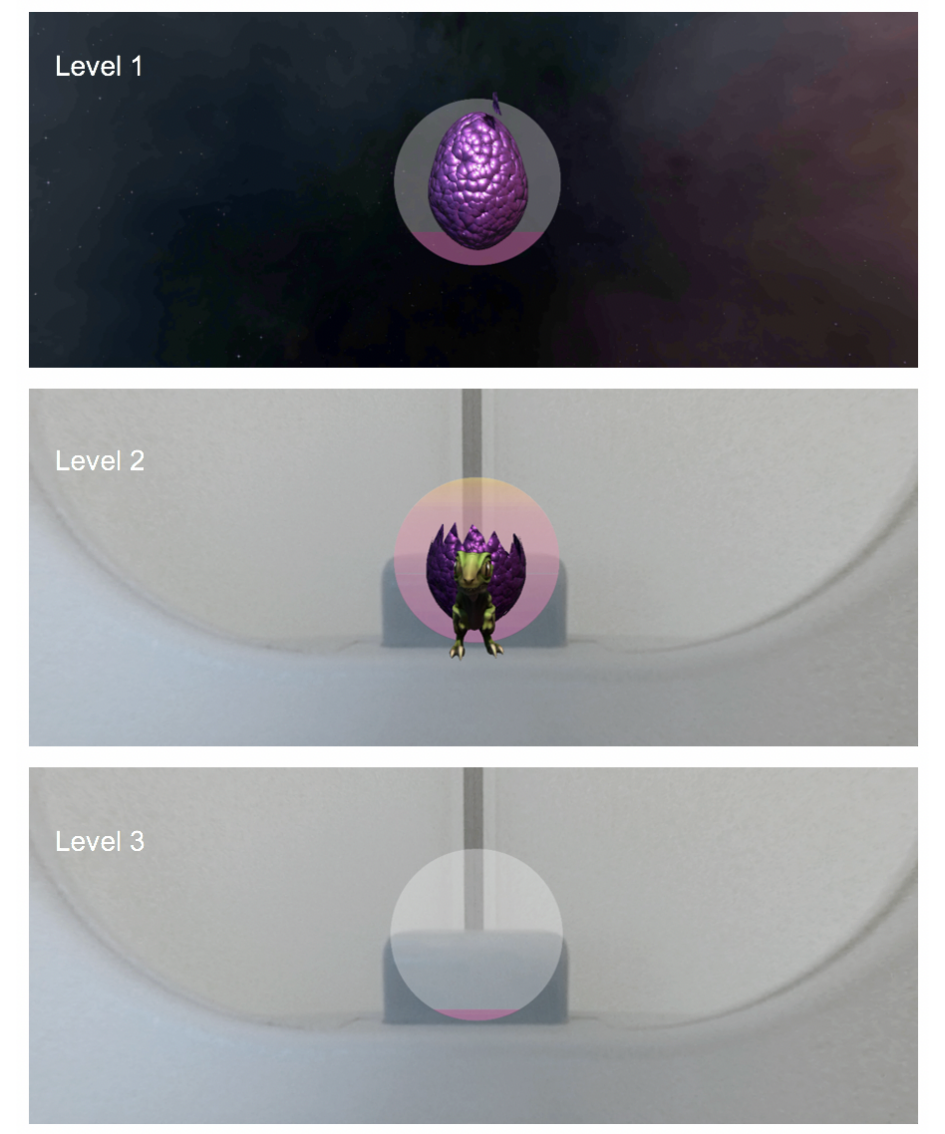
Screenshots of the levels where feedback mechanisms are eliminated and presence in the bore is reintroduced through sounds and visual cues.

As a means of progression, the game has three levels that become more challenging as feedback mechanisms are eliminated, and presence in the bore is reintroduced through sounds and visual cues (Figure 9). The time to complete each game differs between participants, as success can only be achieved by staying still. After the third level was achieved, users were invited to try again by staying still or exit the bore. Once the system was prompted to exit, the user was removed from the tunnel, and the experience was then noted as complete.

### Standard Preparatory Manual

The SPM group received the hospital’s SPM for nonsedated MRI. The manual contains a series of photos showing the MRI experience step-by-step and is intended to help children and their families prepare for medical imaging (Multimedia Appendix 1). Caregivers were instructed to use the manual to prepare for an MRI as they would at home; however, standardized preparation was not enforced as this would not have been reflective of the environment. Materials (eg, chair and MRI sounds) referenced in the manual for practicing were provided.

### Child Life Program

The CLP group received conventional care, where a CCLS was introduced and prepared the participant with the hospital MRI simulator. The curriculum focused on developing rapport with medical professionals, getting comfortable with the hospital setting and medical equipment, assessing reactions to pictures of a real MRI, discussing the upcoming medical procedure, and getting comfortable with earplugs, headphones, loud noises, restraints, the head coil, going into and remaining inside the bore, and holding still. Preparation was not standardized as it would not have been reflective of the current environment. Individualization and adaptation are the tenets of CLP processes.

### Principal Objectives

**Figure 10:**
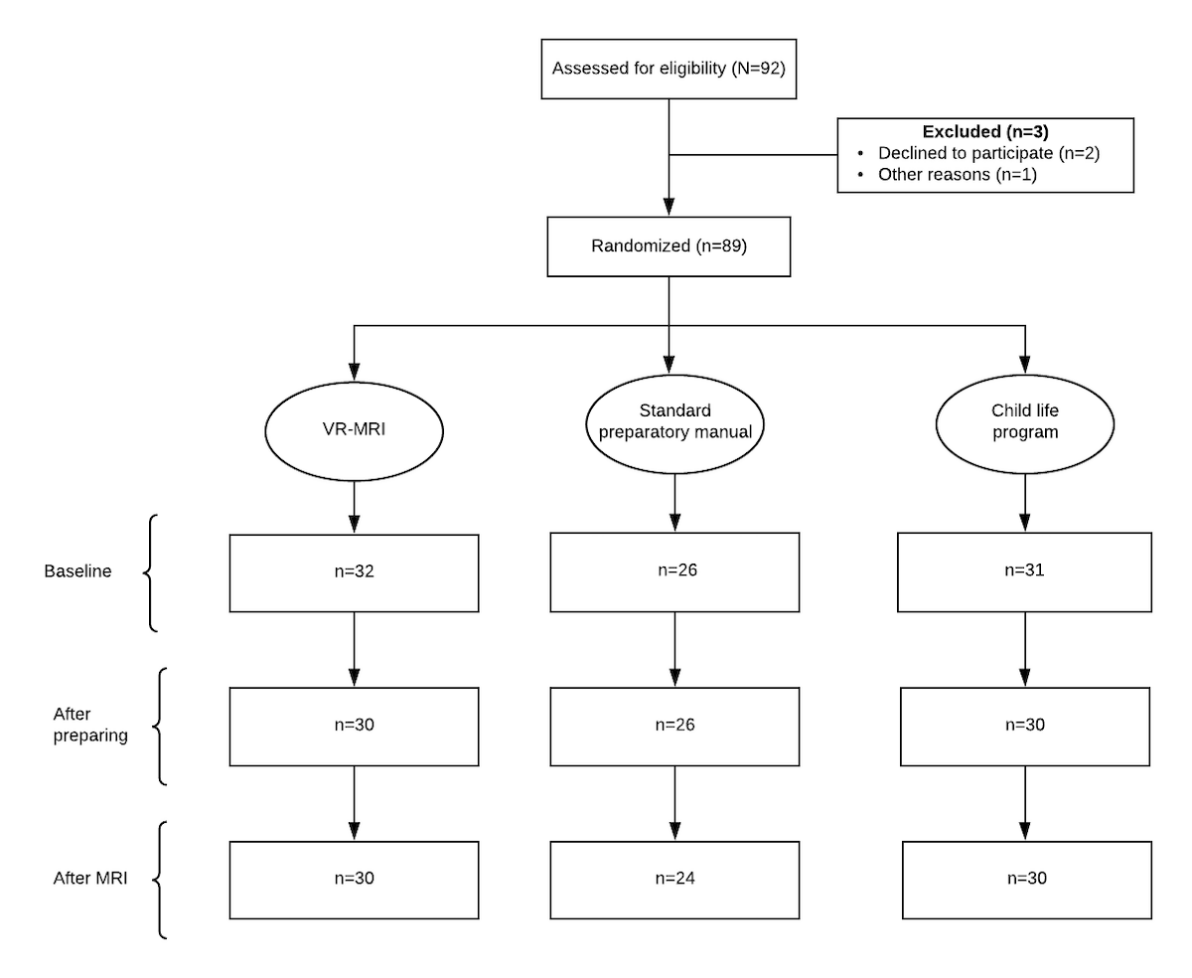
Flow diagram for virtual reality magnetic resonance imaging study. MRI: magnetic resonance imaging; VR-MRI: virtual reality magnetic resonance imaging.

The primary objectives were to evaluate the effectiveness of VR-MRI in preparing children for the MRI simulator experience and reduce child-reported procedural anxiety compared with conventional methods. As a secondary aim, we evaluated caregivers’ reported anxiety, procedural data, parental usability, and child-reported satisfaction with all the preparation materials. Data were collected at 3 distinct timepoints (Figure 10).

### Participants, Randomization, and Procedures

A nonblinded, triple-arm randomized clinical trial was performed at a large provincial hospital in Vancouver (British Columbia), between July 2019 and February 2020. Ethics approval was granted by the University of British Columbia Children’s & Women’s Research Ethics Board (#H19-00371), and the study was prospectively registered at the US National Library of Medicine (#NCT03931382).

The participants were aged 4-13 years. Participants were excluded from the study if they had mental disability, current concussion, significant visual or auditory impairment, inability to speak and understand English, history of seizures or epilepsy, facial or head wounds, or inability to move their head in all directions. All children provided assent, and caregivers or legal guardians provided written consent. Participants received Can $20 (US $16) and parking remuneration.

**Figure 11:**
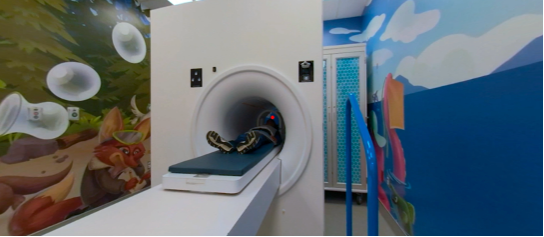
The magnetic resonance imaging simulation room.

Participants were recruited through posters at the hospital, public libraries, community centers, and through social media. We assigned participants in the ratio 1:1:1 to VR-MRI, CLP and SPM and then tested for compliance during a simulated 6-minute MRI scan of the head, designed to replicate an authentic scanning environment (Figure 11). Blinding was not feasible.

### Outcome Measurements

#### Primary Outcomes

The primary outcomes were success in the MRI simulation experience and child anxiety.

#### Success in the MRI Simulation Experience

Movement in the MRI simulation was captured by fitting participants with a motion sensor headband (MoTrak System 1.0, Psychology Software Tools Inc). The sensor system is susceptible to movements of the muscles of facial expression, and has been proposed as one of the most accurate ways to measure the movement of pediatric patients in the absence of estimates from actual MRI [41,42]. Head movement was collected at 8 samples per second, yielding approximately 3300 data points per axis per participant. The threshold for a *successful* MRI, as defined by the department of radiology, is approximately 3-4 mm. Consequently, if at any point during the scan the participant moved >4 mm of cumulative displacement, it was noted as a *fail*.

#### Child Anxiety

Child anxiety was measured with the Venham picture test (VPT; score 0-8) [43]. The VPT has been validated with children for assessing situational anxiety during medical procedures [44] and has a moderately high retest reliability of 0.70 and a high degree of internal consistency with a coefficient α of .838 [43]. Children completed the assessment at three timepoints (before preparing [T1], after preparing and upon entering the simulation room for their 6-minute *scan* [T2], and after the assessment [T3]). The level of the patient’s anxiety was classified as *anxiety-free* (score 0), *low anxiety* (scores 1-3), *middle anxiety* (scores 4-6), and *high anxiety* (scores 7-8).

#### Secondary Outcomes

The secondary outcomes included caregiver anxiety, procedural data, parental usability, child satisfaction, and fun. We also administered a baseline survey to collect information about demographics and clinical characteristics predicted to influence or confound outcomes, such as previous experience with medical imaging.

#### Caregiver Anxiety

Caregiver anxiety was measured with the short State-Trait Anxiety Inventory (STAI; score 6-24), a 6-item, adapted version of the well-validated Spielberger STAI Scale [45]. This measure has a moderately high retest reliability ranging from 0.65-0.75 and a high degree of internal consistency with a coefficient α of .86-.95 [45]. Parents or caregivers were asked to complete the assessment on a tablet at the same time as children (before preparing [T1], after preparing and upon entering the simulation room for their child’s 6-minute *scan* [T2], and after the assessment [T3]).

#### Procedural Data

Procedural data included preparation and assessment times. In accordance with the experiential learning theory [37], we postulated that children who prepared for longer would have a more efficient assessment. Side effects during preparation (eg, nausea and headaches), dropouts, and noncompliance were also recorded.

The *preparation time* started when the study staff finished describing the preparation program, and the researcher indicated that it was time to begin the active preparation. The preparation time was stopped upon indication that the participant felt ready to take the assessment. A maximum preparation time of 45 minutes was allowed. This time frame was selected as it was the allotted appointment time provided by our hospital CLP to prepare patients and their families for medical imaging procedures.

The *assessment time* was defined as the time spent in the simulated MRI room until the participant was *discharged* from the simulation experience, either as successful or noncompliant. A maximum assessment time of 20 minutes was allowed. The time spent transitioning between activities or breaks required for reasons unrelated to the study was not accounted as time.

#### Caregiver Usability

The caregivers of our participants were prompted to provide usability feedback on the preparation materials at the end of the study. Caregivers were asked to complete the USE (Usefulness, Satisfaction, and Ease of Use) Questionnaire administered at the end of the study activities on a tablet. The USE Questionnaire is a 7-point Likert rating scale and is a validated and reliable measure for assessing the subjective usability of a product or service [46]. We limited our testing to the strongest factors of the survey [47].

In addition to this, we asked caregivers if they would be comfortable with their child using the preparation programs and if they had any recommendations for improvement.

#### Child Satisfaction and Fun

For measuring child satisfaction, participants were asked to indicate how satisfied they were with the preparation program by pointing to a visual analog scale ranging from 0 (terrible) to 100 (fantastic). After this, we asked the children if they would recommend the preparation to a friend who needed an MRI and if there was anything that would make their experience better.

Fun was measured using the Smilyometer Likert Scale, a part of the Fun Toolkit [48]. We selected this as a surrogate measure to inform potential adherence and uptake in the real world, assuming that fun would influence use. The Smileyometer was used before and after the children interacted with a preparation program. The rationale for using it before is that it can measure their expectations and for using it afterward is that it is assumed that the child is reporting experienced fun. It has been widely adopted in testing technologies with children to measure satisfaction and fun as it requires no writing [48-52]. After allocation to a preparatory program, children were asked how good they thought the preparation would be by pointing to the Smilyometer Likert Scale to indicate their expectation of using the intervention. After using the preparation and completing the assessment (timepoint 3 [T3]), the children were again asked how good they thought the preparation actually was using the same Smileyometer Likert scale.

### Data Analysis

#### Power Calculation

A priori power analysis was performed using G*Power 3 [53]. Assuming a small-to-moderate effect size (Cohen ƒ=0.20) with 90% power and the probability of a type 1 error of 0.1, a total sample size of 69 was needed (23 in each group).

#### Statistical Analysis

Statistical analysis was performed using SPSS (version 22, IBM Corp). Continuous variables were expressed as mean (95% CI) and ordinal variables as median (IQR). Categorical variables were expressed as percentages. Normality conditions were checked for all variables to apply a proper test of significance. Many of the outcome variables were ordinal in nature and were measured in scores. A chi-square test was used to test the independence of association between categorical variables. Analysis of variance (ANOVA; for normal distribution) or the Kruskal–Wallis test (for nonparametric distribution) was applied for 1-way analysis to compare the average scores of the three interventions among the three timepoints. Post hoc Bonferroni analysis was applied to statistically significant findings to confirm the differences between the groups. If the equal variance assumption was not met during the ANOVA process, pairwise comparisons were based on the statistics of Dunnett T3 [54]. To test for relationships between 2 continuous variables, we used the bivariate Pearson correlation. In the case of missing values, a single value was filled for each missing value by averaging the collected scores for each participant.

## Results

### Demographic and Clinical Characteristics

A total of 92 participants were recruited during the study period; 1 did not consent, and 1 participant did not show up for the appointment. One participant who provided consent initially later withdrew. A total of 89 participants were enrolled. Of the consenting participants, 5% (5/92) were excluded because of equipment malfunction. The remaining 84 participants were included in the analysis (VR-MRI: 30/84, 36%; SPM: 24/84, 29%; CLP: 30/84, 36%).

The demographic and clinical characteristics of the patients are shown in Table 1. Most participants were male (51/84 61%) and had no history of MRI (77/84, 92%) or simulator experience (81/84, 96%). Approximately half the participants (43/84, 51%) had experience with other medical imaging procedures, and many had used VR before participating in the study (70/84, 83%). Chi-square tests were conducted for demographic variables and ANOVA for continuous variables. No significant differences in demographic variables were found among the groups (Table 1).

**Table 1:** Demographics and clinical characteristics of participants (N=84).

Characteristics		Total population (n=84)	VR-MRI^a^ (n=30)	Standard preparatory manual (n=24)	Child Life Program (n=30)	*P* value
Age (years), mean (SD)		9.1 (2.7)	9.3 (2.6)	8.9 (2.8)	9.2 (2.7)	.51
Males, n (%)		51 (61)	18 (60)	13 (54)	20 (67)	.64
**History with, n (%)**						
	Magnetic resonance imaging	7 (8)	3 (10)	3 (12)	1 (3)	.44
	Any other medical imaging	43 (51)	13 (43)	12 (50)	15 (50)	.84
	Magnetic resonance imaging simulator	3 (4)	2 (7)	—^b^	1 (3)	.42
	Virtual reality	70 (83)	24 (80)	18 (75)	25 (83)	.75

^a^VR-MRI: virtual reality magnetic resonance imaging.

^b^Not available.

### Success in the MRI Simulation Experience

Success was indicated if participants were able to complete a 6-minute head scan without surpassing 4 mm of movement at any of the 3300 data points collected. The average number of times participants scored above the threshold for the 3300 data points was not statistically significant between the groups (*χ*^2^_2_=2.7; *P=*.07). Similarly, no statistically significant differences were found when calculating success in the simulated MRI experience among VR-MRI, SPM, and CLP (*χ*^2^_2_=2.6; *P=*.27). On average, 30% (95% CI 13%-47%) participants in the VR-MRI group were successful, compared to 50% (95% CI 28%-72%) in the SPM group and 47% (95% CI 28%-66%) in the CLP group. Of the participants who failed, 8 (VR-MRI: 2/8, 25%; SPM: 3/8, 37%; CLS: 2/8, 25%) were noncompliant by declining to complete some or all of the requirements of the scan, automatically failing their assessment.

### Child Anxiety

**Figure 12:**
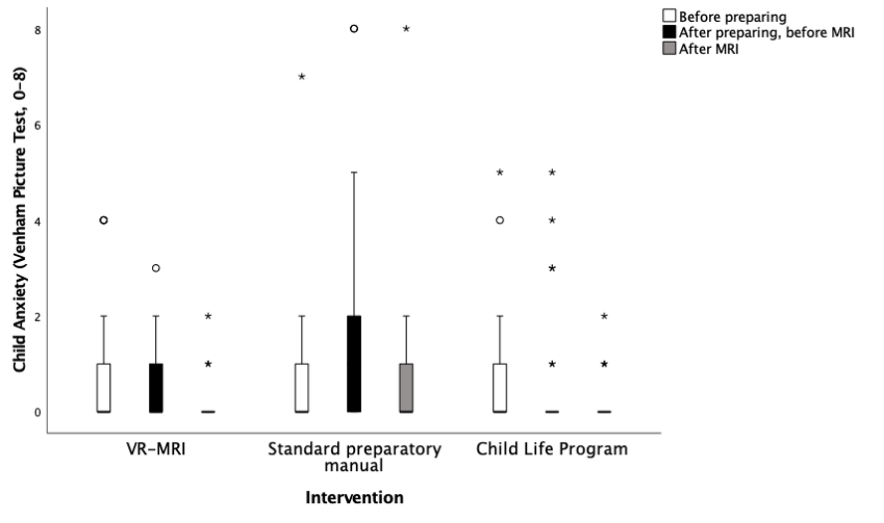
Comparison of self-reported child anxiety across groups and timepoints, measured by the Venham picture test (the circles denote outliers and the asterisks denote extreme outliers). No clinically significant results were indicated (timepoint 1, *P*=.99; timepoint 2, *P*=.008; timepoint 3, *P*=.10). MRI: magnetic resonance imaging; VR-MRI: virtual reality magnetic resonance imaging.

VPT was used to determine how anxious children were before preparing (timepoint 1 [T1]), after preparing and upon entering the MRI simulation room (timepoint 2 [T2]), and after completing the assessment [T3]). Participants reported that anxiety remained relatively stable, with no *clinically* significant differences between the groups at any timepoint (Figure 12; T1, *P*=.99; T2, *P*=.008; T3, *P*=.10). On average, children in the VR-MRI group reported being anxiety-free before preparation (median 0, IQR 1; SD 1.311), after preparation (median 0, IQR 1; SD 0.819), and after the assessment (median 0, IQR 1; SD 0.434). Children in the SPM group reported being anxiety-free before preparation (median 0, IQR 1; SD 1.521), having low anxiety after preparation (median 1, IQR 2; SD 2.311), and being anxiety-free again after the assessment (median 0, IQR 1; SD 1.738). Finally, children in the CLP group reported being anxiety-free before preparation (median 0, IQR 0; SD 1.240), after preparation (median 0, IQR 0; SD 1.350), and after the assessment (median 0, IQR 0; SD 0.468).

### Caregiver Anxiety

**Figure 13:**
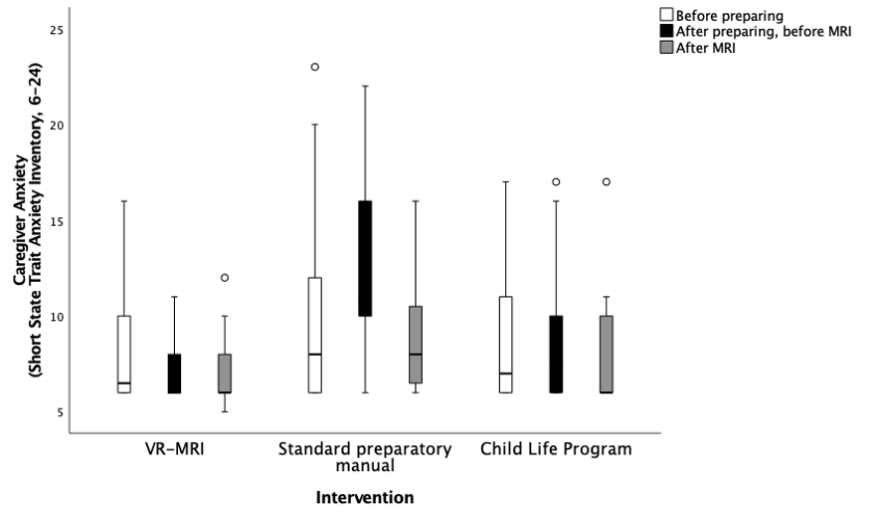
Comparison of change in self-reported caregiver anxiety between timepoints and across groups, measured with the short State-Trait Anxiety Inventory (the circles denote outliers and the asterisks denote extreme outliers). Clinically significant differences were found between the manual group in comparison with the virtual reality magnetic resonance imaging (Dunnett *P*<.001) and the Child Life Program (Dunnett *P*=.004). MRI: magnetic resonance imaging; VR-MRI: virtual reality magnetic resonance imaging.

The short STAI was used to determine how anxious caregivers were before preparing (T1), after preparing and upon entering the MRI simulation room (T2), and after completing the assessment (T3). As depicted in Figure 13, caregivers using the manual to prepare were significantly more anxious after preparing than caregivers in the VR-MRI (mean difference 5.33, 95% CI 2.93-7.74; Dunnett *P*<.001) and CLP groups (mean difference 3.73, 95% CI 1.07-6.40; Dunnett *P*=.004). The effect size was large (η^2^=0.319, 95% CI 0.15-0.448). No clinically significant differences between the groups were found before preparing (T1) or after completing the assessment (T3). Caregivers in the VR-MRI group reported low anxiety before (median 6.5, IQR 4; SD 2.572) and after preparation (median 6, IQR 2; SD 1.744) and after the assessment (median 6, IQR 2; SD 1.810). Caregivers in the manual group reported low anxiety before preparation (median 8, IQR 6; SD 4.945), an increase in anxiety after preparation (median 10, IQR 6; SD 4.394), and again low anxiety after the assessment (median 8, IQR 4; SD 2.924). Finally, caregivers in the CLP group reported low anxiety before (median 7, IQR 6; SD 3.294) and after preparation (median 8, IQR 4; SD 3.224) and after the assessment (median 6, IQR 4; SD 2.545).

We conducted a bivariate Pearson correlation analysis to determine whether child anxiety was related to caregiver anxiety. After preparation and upon entering the MRI simulator room, child and caregiver anxiety had a statistically significant linear relationship (r_84_=0.421; *P*<.001), indicating that child anxiety increased with parental anxiety. The strength of this relationship was moderate.

### Procedural Data

Time was recorded during preparation and assessment. There was a significant difference in preparation times among all groups (*F*_2.81_=53.261; Dunnett *P*<.001). The effect size was large (η^2^=0.568, 95% CI 0.415-0.660). Participants in the VR-MRI group prepared for the longest time, which was 22.05 minutes (SD 4.41; 95% CI 20.40-23.69), compared with the CLP group, which was in the middle at 15.06 minutes (SD 3.32; 95% CI 13.82-16.30), and the SPM group, which prepared for the shortest time, at 9.98 minutes (SD 5.24; 95% CI 7.76-12.20).

No statistically significant differences were found among the groups in assessment time (*F*_2.81_=2.063; *P=*.13). Participants in the VR-MRI group, on average, took 11.79 minutes (SD 1.99; 95% CI 11.05-12.55), compared with the SPM group who took 11.19 minutes (SD 3.80; 95% CI 9.58-12.78) and the CLP group who took 10.17 minutes (SD 3.44; 95% 8.89-11.45) to complete their assessment (regardless of success).

**Figure 14:**
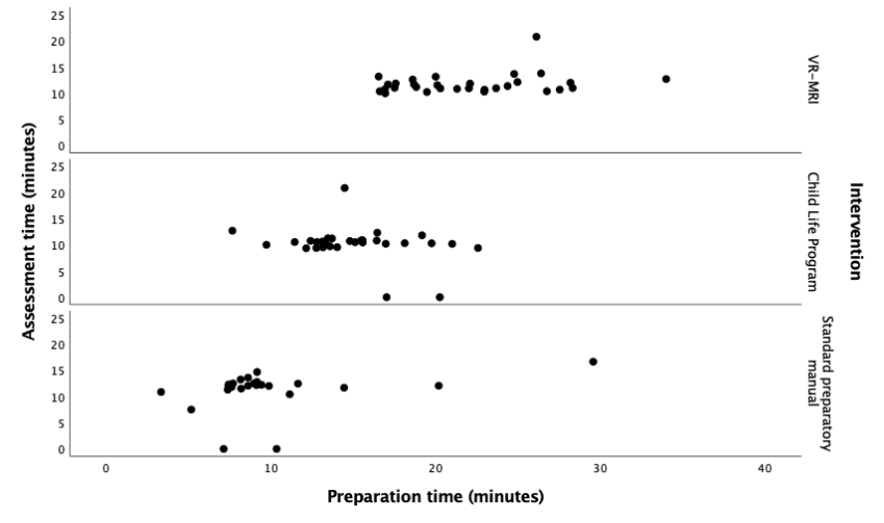
Comparison of preparation and assessment times across groups, measured in minutes. No significant relationship was found (r=0.148; *P*=.18). VR-MRI: virtual reality magnetic resonance imaging.

We conducted a bivariate Pearson correlation test to determine whether the time spent preparing affected the efficiency of the assessment. Preparation and assessment times did not have a statistically significant linear relationship (r=0.148; *P*=.18), indicating that preparation time did not have a significant effect on assessment time in our study (Figure 14).

One child (aged 5 years) indicated eye strain and a blurry image when viewing VR-MRI that could not be mitigated by interpupillary adjustments. Two children (aged 6 and 4 years) reported the dinosaur graphic in VR-MRI was *scary*. Six participants (aged 4-12 years) in the manual group expressed being scared of pictures in the manual, particularly the sections of the intravenous or coil pictures. No other side effects were reported.

### Caregiver Usability

**Figure 15:**
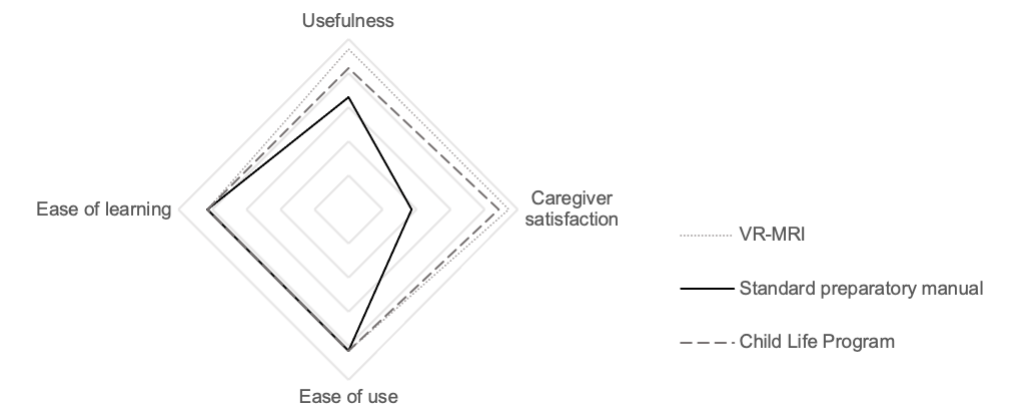
Comparison of median usability metrics collected across groups, adjusted to total scores of 100. Significant differences were found for caregiver satisfaction when comparing the manual with the virtual reality magnetic resonance imaging and Child Life Program (Bonferroni *P*<.001). VR-MRI: virtual reality magnetic resonance imaging.

Caregivers were asked to complete the USE Questionnaire [46] to report how easy the preparation materials were to learn and use, as well as how useful and satisfied they were with using them to prepare their children for the simulated MRI experience. Caregivers did not report significant differences in ease of use (*χ*^2^_2_=0.01; *P*=.99), ease of learning (*χ*^2^_2_=1.5; *P*=.48), or usefulness (*χ*^2^_2_=4.4; *P*=.11) among the groups. On average, caregivers using VR-MRI to prepare their child agreed that it was useful (median 31, IQR 4; SD 3.562), easy to use (median 24, IQR 3; SD 2.448), and easy to learn (median 18, IQR 2; SD 2.366). As depicted in Figure 15, caregivers using CLP, on average, also *agreed* that it was useful (median 30, IQR 6; SD 4.163), easy to use (median 24, IQR 4; SD 2.937), and easy to learn (median 18, IQR 3; SD 3.059). Caregivers using SPM *somewhat agreed* that it was useful (median 28.5, IQR 6; SD 3.323) and *agreed* that it was easy to use (median 24, IQR 5; SD 2.167) and easy to learn (median 18, IQR 3; SD 2.183).

However, when reporting satisfaction with the preparation materials, caregivers using VR-MRI and CLP were significantly more satisfied than the caregivers using SPM (Dunnett *P*<.001). The effect size was large (η^2^=0.268, 95% CI 0.104-0.402). Caregivers in the VR-MRI (median 31, IQR 4; SD 2.837) and CLP (median 30.5, IQR 6; SD 4.173) groups reported high satisfaction. However, caregivers in the SPM group were only somewhat satisfied (median 26, IQR 6; SD 2.428).

When asked if they would be comfortable with their child using the preparation materials for an actual MRI, 100% of participants in all groups indicated *yes*. In terms of improvements, caregivers in the VR-MRI group indicated they wanted the ability to explore VR-MRI in tandem with their child and wanted more information about when their child would be ready for a successful MRI. Caregivers in the manual group recommended tailoring the manual to the specific procedures being conducted, offering more instruction to parents on how to coach their child through the MRI experience, and offering more interactivity (eg, videos and stickers) to guide children through the steps. Caregivers in the CLP group were pleased and had no recommendations for improvement.

### Child Satisfaction and Fun

Child satisfaction is reported in Table 2. Children were asked to point to a visual analog scale, indicating their satisfaction with the preparation materials. Children in the SPM group were significantly less satisfied than those in the CLP group (mean difference −17.509, 95% CI −34.85 to −0.17; Dunnett *P*=.047). On average, children in the manual group were 73.5% (SD 27%; IQR 37%) satisfied compared with children in the CLP group who were 90% (SD 12%; IQR 23%) satisfied. Children in the VR-MRI group were 80% (SD 27%; IQR 22%) satisfied.

**Table 2:** Comparison of self-reported child satisfaction scores between groups, measured with a visual analog scale.

Characteristics			Participant (n=77), n (%)		Median score (IQR; SD)		Mean rank		Chi-square (*df*)		*P* value
**Timepoint 1**											
	VR-MRI^a^	30 (39)		80 (22; 27)		36.15		7.7 (2)		.02^b^	
	Standard preparatory manual	20 (26)		73.5 (37; 27)		30.90		—^c^		—	
	Child Life Program	27 (35)		90 (23;12)		47.17		—		—	

^a^VR-MRI: virtual reality magnetic resonance imaging.

^b^Statistically significant difference between study arms (Kruskal–Wallis, *P*<.05).

^c^Not available.

The Smileyometer was used to determine how fun children expected the programs to be and how fun they actually were after completing them. We did not find any statistically significant differences in how much fun children expected the preparation to be (*F*_2.81_=2.224; *P*=.11). On average, the children in the VR-MRI and CLP groups thought the preparation would be *really good*, whereas children in the manual group thought the preparation would be *okay*.

When asked if they would recommend the preparation materials to a friend, 93% (28/30) of participants in the VR-MRI group said yes compared with 80% (22/27) in the CLP group and only 71% (14/20) in the manual group. Participants in the VR-MRI group thought the game was too difficult and that they spent too much time staying still. The participants reported that they wanted more options in terms of selecting a character in the experience and tailoring the language to their age group. Participants in the manual group recommended only including the procedures they would encounter and providing more interactivity. The participants in the CLP group also commented on the length of time required to stay still and the importance of listening to the correct volume and repetition of the sounds.

## Discussion

### Effectiveness of VR-MRI in Preparing Children for the Nonsedated MRI Experience

In this study, we compared a VR preparation program (VR-MRI) with the hospital CLP and SPM for reducing anxiety and noncompliance during a simulated MRI scan of the head. To our knowledge, no other study has compared VR with these alternatives for nonsedated MRI using both anxiety and success as outcomes.

Although VR has been studied in some preprocedural situations, including anesthesiology and medical imaging, its effectiveness in comparison with mock-MRIs [15,16,55-57] and other behavioral techniques [4,12] used by CLPs has not been well examined [12]. We found no significant differences between VR-MRI, our hospital CLP, and our hospital SPM on our measure of success during a simulated MRI experience (*P=*.27). This finding is comparable with that of another recent study [56] which found cheaper versions of MRI simulations adequate in most cases to provide the desensitization and practice needed for a successful nonsedated pediatric MRI scan. VR-MRI adds to the options for affordable and viable alternatives discussed in recent reviews [12]. It can be used at home or in remote settings for further practice and can easily be stored when not in use. Thus, health care centers could use a digital preparatory sequence leveraging immersive media, such as our VR-MRI app, to prepare pediatric patients aged between 4 and 13 years without the financial, travel, and space requirements of traditional in-person MRI simulations.

### VR-MRI and Child Anxiety

In addition to finding no differences in our measure of success, we also found no clinically significant differences among children’s reported anxiety when using VR-MRI compared with the other standards of care (T1, *P*=.99; T2, *P*=.008; T3, *P*=.10). Other studies have reported low anxiety during medical imaging simulations [30]. The findings of this study and ours question the relationship suggested by many studies (that anxiety directly influences motion artifacts). Although our study participants reported low anxiety, many were still unsuccessful in the simulated MRI experience. A patient’s understanding is another element that can contribute to motion artifacts [58]. However, our findings also question this as we did not find any relationship between success and prior experience with medical imaging. Our sample for this subset was quite small, which could explain why no relationship was found. Future studies could consider measuring heart rate and cortisol as objective measures of stress throughout the training and procedure, as well as a measure of patient understanding to further explore these concepts.

### VR-MRI and Caregiver Anxiety

Whereas other research has focused primarily on patient anxiety, our study uniquely evaluated caregivers' anxiety during the process of preparing for and completing a simulated MRI experience. VR-MRI was not statistically different from CLP and was better than SPM in mitigating caregiver-reported anxiety when entering the MRI simulation room (*P*<.001). We also found that caregiver and child anxiety tended to increase together after preparation (*P*<.001). Given that it is generally accepted that clinicians provide the opportunity for parents to be present during their child’s procedure [59], our study results suggest that preparation is just as beneficial to caregivers as it is to the children undergoing MRI procedures. To assist in caregiver preparation, we mirrored the experience on a tablet for parents to experience it at the same time as the children. To our knowledge, effective ways of engaging caregivers in the preparation process have not yet been studied, particularly in the context of VR.

### Time Spent Preparing and Conducting Head Scans

Participants prepared the longest with VR-MRI and the shortest with SPM. This was largely expected given that VR-MRI was a standardized experience compared with the experience of the other two groups that received interventions tailored to the participant by the CCLS (in the CLP group) or the caregiver (in the SPM group). It is interesting to note that participants engaged in programs that provided opportunities for experiential learning (VR-MRI and CLP) for a longer duration than those that offered through didactic learning (SPM). However, these novel elements of preparation, which allowed patients to engage and participate in a practice MRI experience, did not result in differences in success. We did not find any significant differences in the assessment times across the groups (*P=*.13).

We did not continue to conduct training and assessments with participants until we obtained an appropriate image quality, which would be critical for clinical care scenarios. In fact, many of the children failed the assessments in our study, suggesting that the assessment was actually not efficient, and more training or sedation would be required for these children. An important element of experiential learning is the opportunity to reflect on experiences [37]. As our study comprised a single session on the same day as the assessment, and children aged ≥6 years benefit most if they participate in preparation programs for ≥5 days in advance of the procedure [4], offering VR preparation before a medical imaging procedure and with multiple opportunities to experience, reflect, and practice should be explored by future investigators. In accordance with patient-centered care, children may also benefit from interacting with different practice materials depending on their individual preferences. We echo other investigators in stating that deploying multiple strategies together may provide the best way forward to improve nonsedated medical imaging outcomes and experiences [12].

### Experiences With VR-MRI

#### Caregivers’ Experiences

As reported by other investigators [27,29-31], experiences using VR-MRI were positive in our study. Our data indicate that compared with CLP and SPM, the VR-MRI app was just as easy to use (*P*=.99), easy to learn (*P*=.48), and useful (*P*=.11). The technical concerns commonly reported as barriers to perceived ease of use of VR technologies [60] were not mentioned by the caregivers in our study. In fact, caregivers were significantly more satisfied with VR-MRI than SPM (*P*<.001). It is important to note that SPM was the only preparation program to require support from the caregiver as the researcher assisted in the setup for the VR-MRI group, and the CCLS assisted in the CLP group. As such, we may not have seen the same result if participants were required to set up the hardware themselves without assistance. Nonetheless, VR-MRI was perceived as a valued and acceptable form of preparation by caregivers.

#### Children’s Experiences

In addition to caregiver experiences, we also measured the children’s experiences using VR to prepare for their simulated head scan. We only found significant differences in children’s reported satisfaction between SPM and CLP (*P*<.001) groups. Children using the VR-MRI app generally thought it was *really good* for preparing them for the MRI assessment, meeting the children’s expectations for fun. The results of the app meeting expectations are likely influenced by the VR-MRI design, which includes obtaining multiple perspectives for identifying the needs and values of pediatric patients undergoing medical imaging procedures [34,35]. However, it is interesting that there were no significant differences among VR-MRI and the other 2 groups in terms of fun. Other investigators using gamification in VR for preoperative anxiety in pediatric patients undergoing general anesthesia also found that satisfaction was not significantly different between their gamified and control groups [24]. The result suggests that integrating novel technologies does not inherently make a procedural preparation *fun*, and further product and design elements are required to create an optimal experience. On the basis of the feedback from children and their caregivers, the VR-MRI game design may need refinements in terms of tailoring the challenge of staying still to the user’s initial skills, as well as more options for customization and immersion to create an optimal experience [21,34,39].

### Product and Design

The selection of preprocedural scenarios and hardware that are best for integration with VR is not well understood and has not been the focus of previous pediatric studies [21,39]. In our study, we elected to conduct a head scan as it was one of the most common and anxiety-provoking scans at our hospital. However, pragmatically, the clinical characteristics of patients who require head scans may not be the most appropriate for the use of VR headsets as a significant proportion of patients who require head scans have traumatic brain injuries or are potentially epileptic (which typically precludes them from using VR). In addition, experiences could be affected by a patient’s mobility of the head and neck when using HMDs. Further research is needed for safe use with these patients and may include delivering the program through immersive videos rather than with a headset. The program must also be adapted to make it available on other platforms (Android and iOS) and devices so that it can be versatile in deployment [39]. The lack of appropriate patients to recommend VR to had a significant impact on the uptake of VR interventions in other clinical settings [60] and should be an important consideration for development.

Our study responds to the literature calling for studies on products that engage pediatric patients and explore skill-building goals [34]. We introduce intervention and design elements that provide dynamic feedback to the patient and experiential learning to regulate movement in preparation for a nonsedated MRI. We designed and tested a virtual MRI with real-time feedback to explore skill building and introduced some product qualities that enabled the participants to be active in preparing themselves for the MRI experience. Our design considerations included tailoring simplicity and interactivity to improve control, improving a sense of presence, creating a sense of safety through familiar design elements and medical procedures, incorporating narrative elements, and cultivating growth and motivation [34]. The real-time feedback feature, facilitated through a mobile phone gyroscope, is a unique gamification element that aligns with anxiety management strategies [34]; to our knowledge, this has not been reported in the literature to date. Future apps should focus on refining and validating real-time feedback gaming elements in medical imaging preparation so that it could be used as a decision-making tool that informs parents and health care providers about when and if a patient might be ready to attempt a nonsedated MRI, thus reducing the burdens associated with anxiety and noncompliance.

### Limitations

There are several potential limitations to this study. Our methodology focused on self-reporting of anxiety of children and caregivers. We used the short STAI for caregivers but VPT for children, which may have introduced confounding factors. The results may also have been affected if users did not fully understand the meaning or how to complete the surveys after the instructions. There may have been a response bias, as children often consciously or subconsciously give responses that they think adults want to hear.

Our study also had several additional biases. The study was subject to information and selection biases, as we recruited participants through posters at the hospital, public libraries, and social media and provided remuneration and parking reimbursements. Motivation and reported outcomes related to using the materials could have been affected by these extrinsic motivations (eg, remuneration). The study was also unblinded to the participants and research staff because of the practicalities of the preparatory processes and logistical limitations.

Our study had a small sample size that met the requirements of our power calculation. The effect sizes were smaller among groups than we anticipated, and, therefore, this study is at risk for type 2 error (accepting a null hypothesis that is actually false). A total of 5 participants did not have movement metrics because of technological malfunction. In our study, we used the MERGE VR headset as no other HMDs have been indicated for use specifically with children. This headset is indicated to match the interpupillary distance of children aged ≥10 years. Younger children may have smaller interpupillary distances than can be adjusted for. Eye strain and a blurry image that could not be mitigated by adjustment were reported by 1 study participant (aged 5 years), which is likely a result of that limitation. Currently available consumer-grade VR hardware has not been designed for use with younger children and, in some cases, might not be adjustable for the parameters required by them.

### Conclusions

The VR preparatory program with a novel real-time feedback feature had comparable findings with our hospital CLP and SPM in successfully preparing children to complete a simulated MRI experience. Furthermore, VR-MRI mitigated situational anxiety in children throughout the process of preparation and completion of a simulated MRI head scan. As such, digital preparatory apps that leverage immersive media, such as our VR-MRI app, may be a viable alternative for preparing children for nonsedated MRI. Further research is required to confirm the findings with actual pediatric patients in a real MRI machine, as our study used a high-fidelity MRI simulator. Nevertheless, the use of VR and the sequence of activities provided through the VR-MRI app show promise for preprocedural anxiety reduction in children and their caregivers.

## Appendix

Multimedia Appendix 1 Standard preparatory manual.

Multimedia Appendix 2 CONSORT-eHEALTH checklist (V 1.6.2).

## Abbreviations

ANOVA: analysis of variance
CCLS: certified child life specialist
CLP: Child Life Program
HMD: head-mounted display
MRI: magnetic resonance imaging
SPM: standard preparatory manual
STAI: State-Trait Anxiety Inventory
T1: timepoint 1
T2: timepoint 2
T3: timepoint 3
USE: Usefulness, Satisfaction, and Ease of Use
VPT: Venham picture test
VR: virtual reality
