# Supplementary material for: Comparing a Virtual Reality–Based Simulation App (VR-MRI) With a Standard Preparatory Manual and Child Life Program for Improving Success and Reducing Anxiety During Pediatric Medical Imaging: Randomized Clinical Trial
Source: J Med Internet Res. 2021 Sep 22;23(9):e22942. doi: 10.2196/22942 (PMC8495586; doi:10.2196/22942)

MRI Preparation Book

For families

BC Children's Hospital

BC Children's Hospital
An agency of the Provincial Health Services Authority

Introduction

This book contains a series of photos showing the MRI experience step-by-step to help prepare you for your MRI.

Magnetic Resonance Imaging (MRI) uses a powerful magnet to take pictures of the inside of your body. The machine makes loud noises as it takes pictures. Some children and youth compare these noises to knocking, an alarm, or running shoes in the dryer.

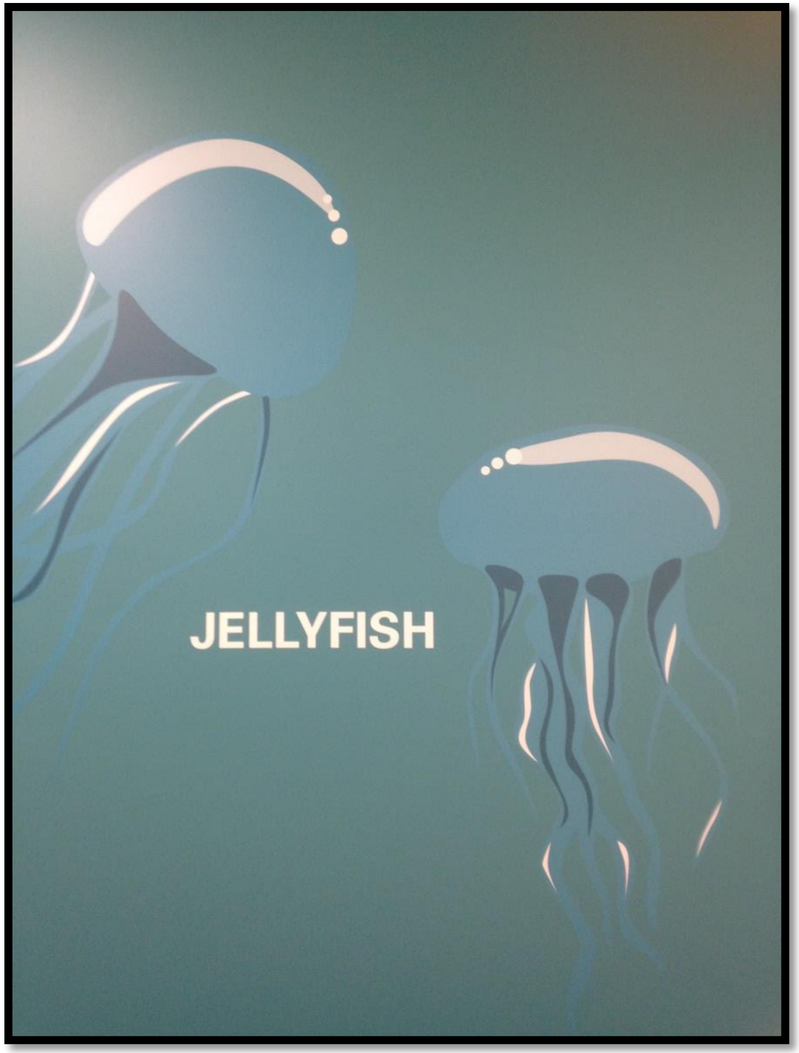

MRI sounds

This is a youtube link to sounds that an MRI machine makes.

http://www.youtube.com/watch?v=9GZvd_4ot04

Still as a statue

Have you ever tried taking a picture of something that is moving? It gets blurry!

It is very important that you stay still for your MRI pictures to make it easier for the doctors to see what is happening inside your body.

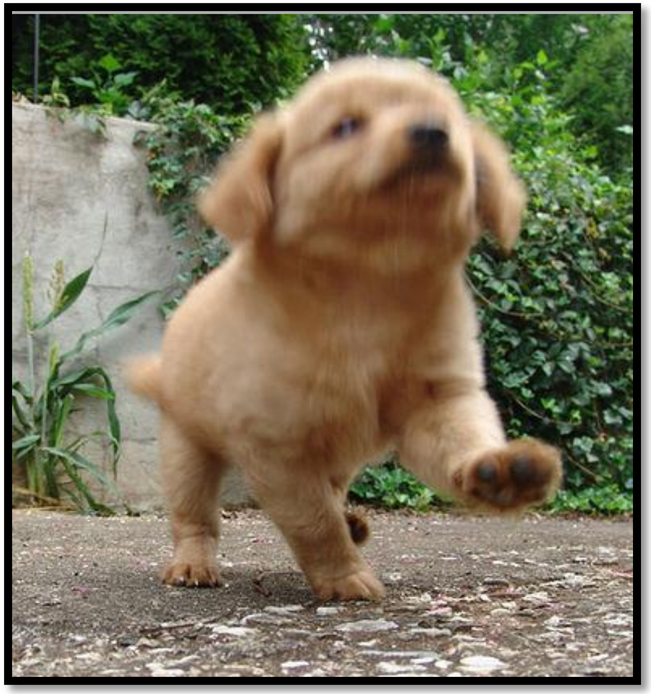

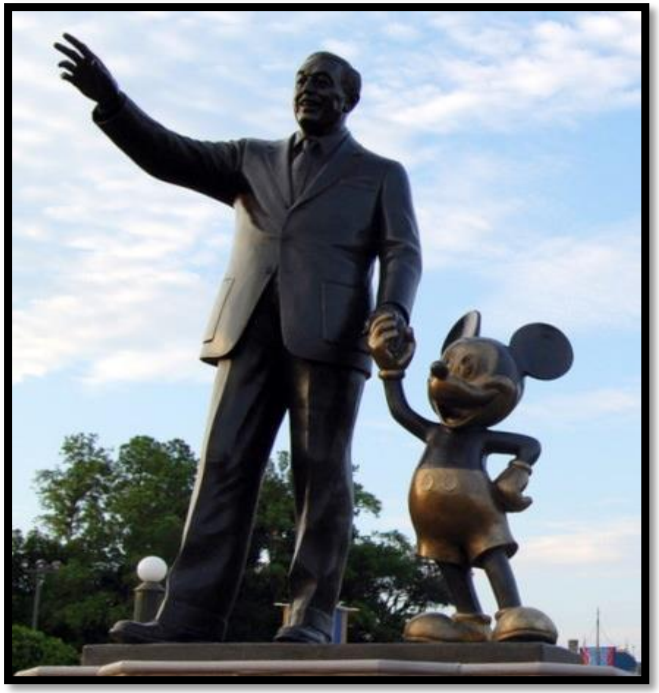

Arrival

When you arrive a nurse will give you hospital pyjamas to wear during your scan.

During your scan one adult from your family can go with you if you want. They will get changed into hospital pyjamas too.

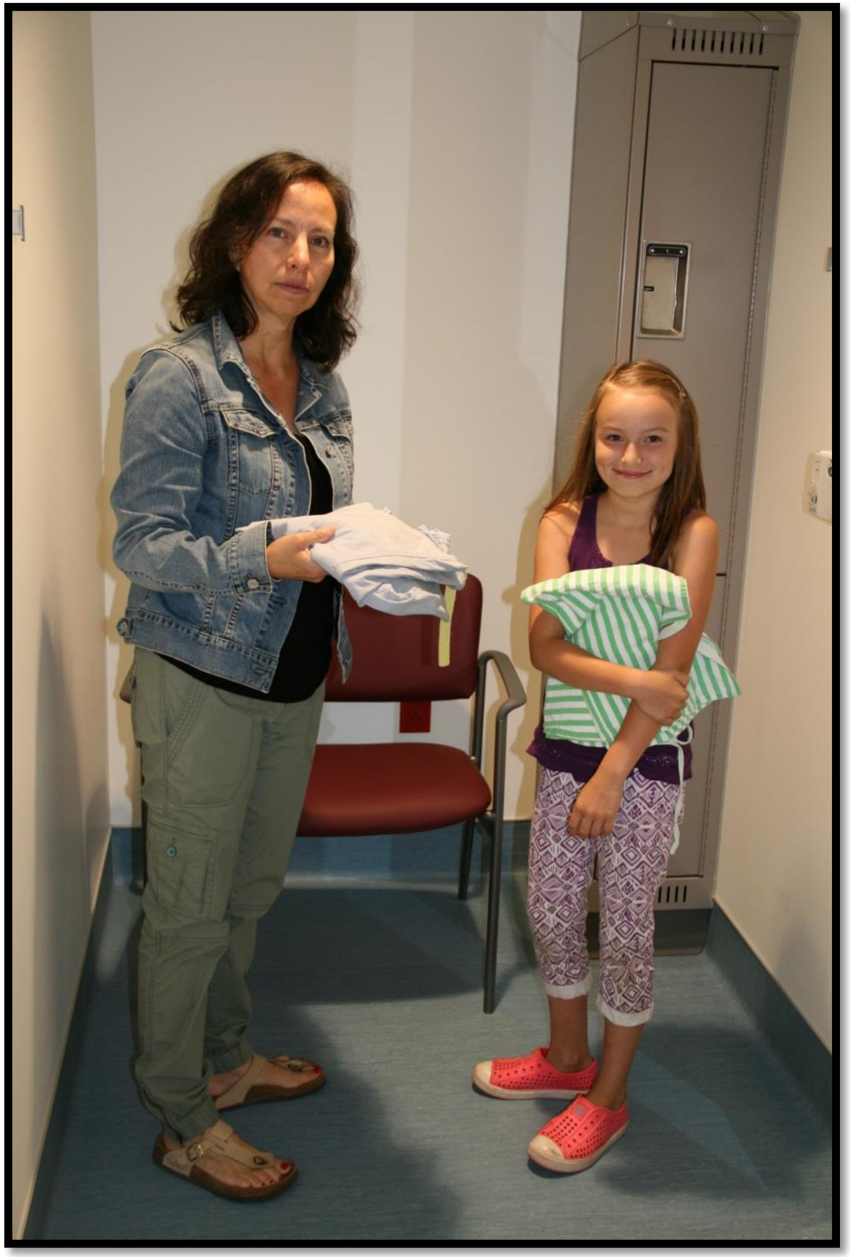

Weight

The nurse will bring you to another room where you will stand on a scale to see how much you weigh.

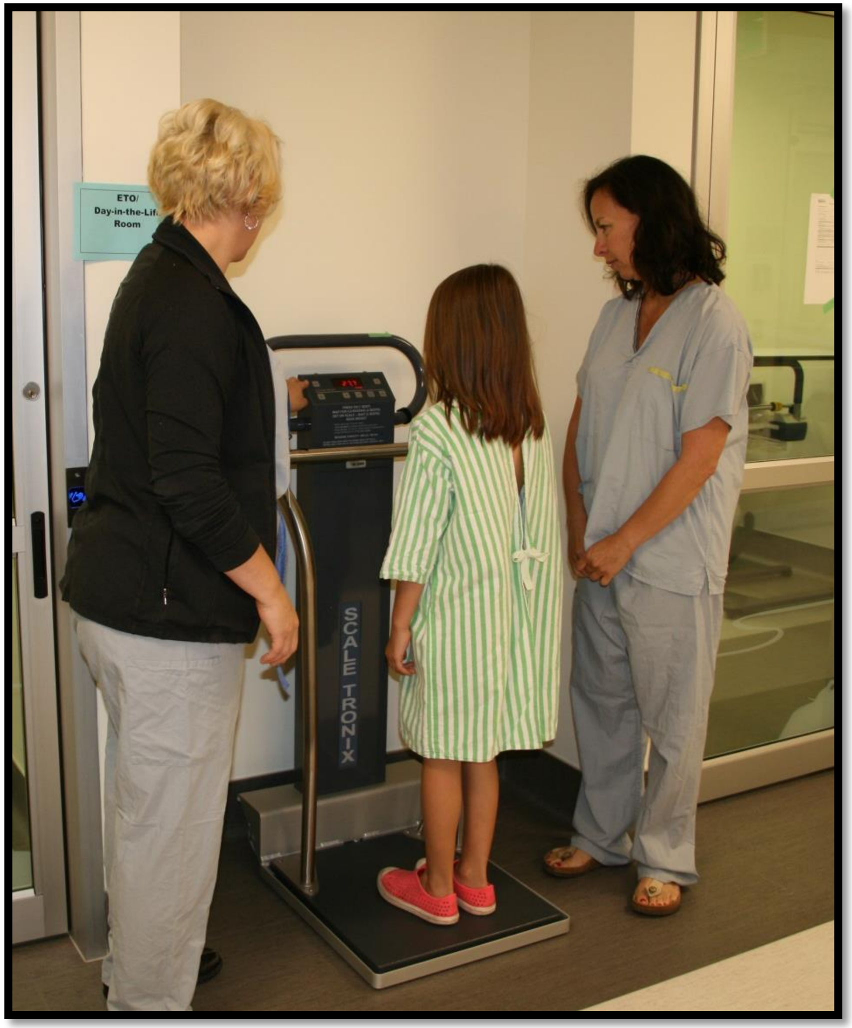

Checking for metal

It is important to remove all metal before your MRI scan. Things like hair clips, jewellery and glasses are not allowed in the MRI room.

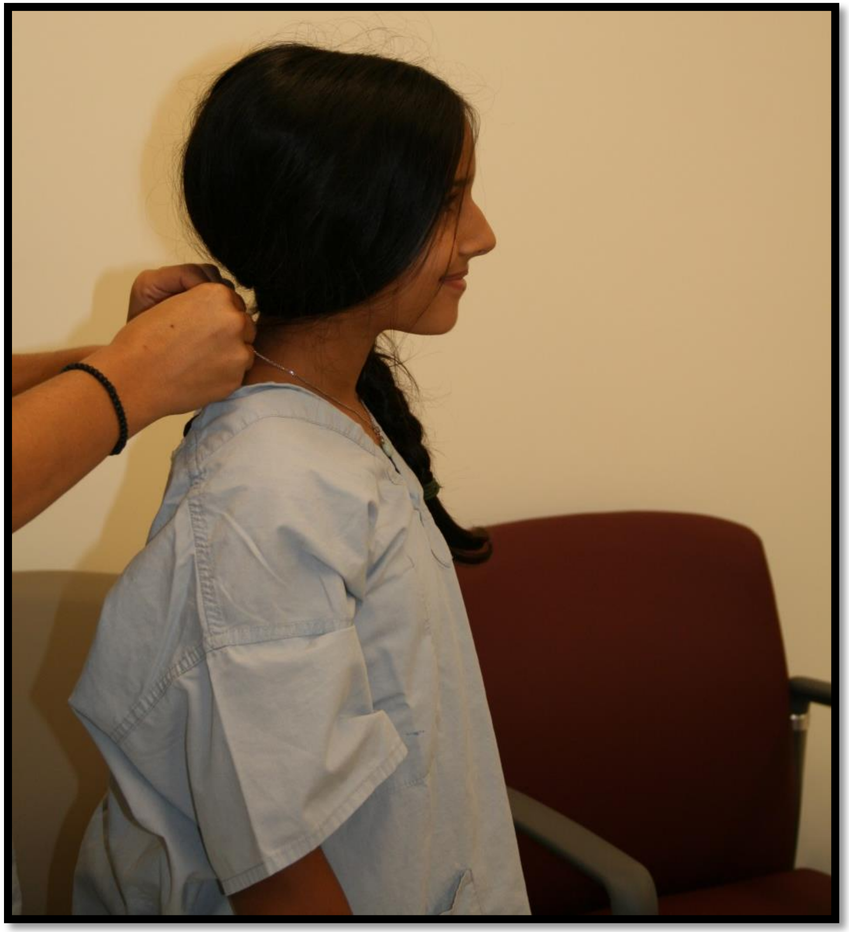

The MRI machine

The MRI machine is a circle shape with a hole in the centre. The back of the camera is open making it a tunnel.

The bed will slide into the hole; the pictures are taken while you are inside the tunnel.

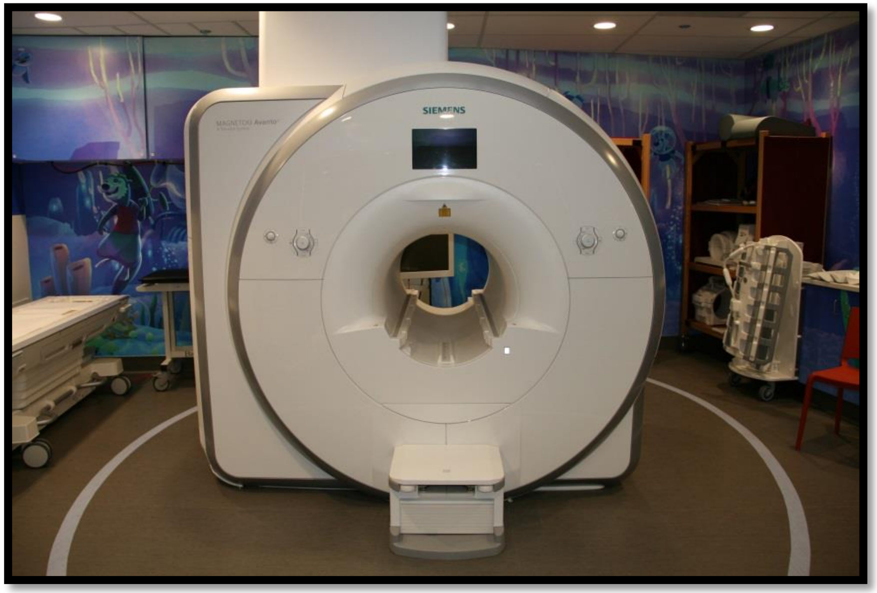

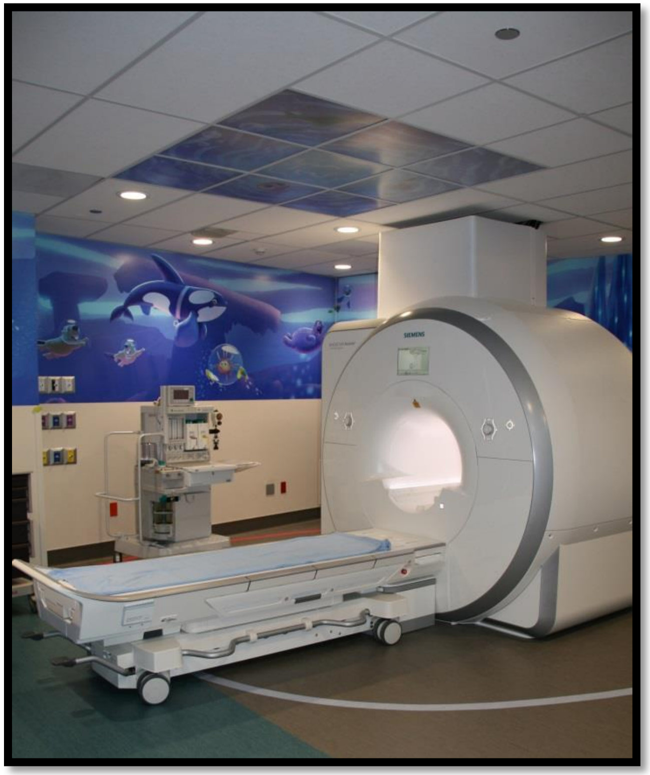

The murals

There are two MRI machines. The murals on the wall look different in each MRI room. You might be in the room with the ocean or the sky theme.

Be sure to look up to see the art on the ceiling too!

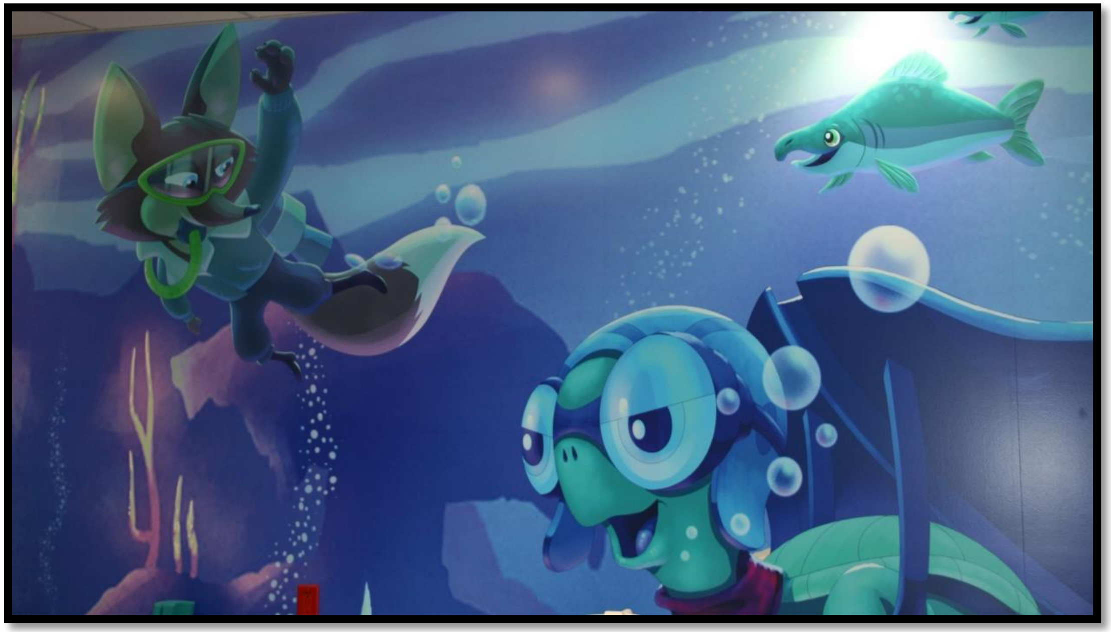

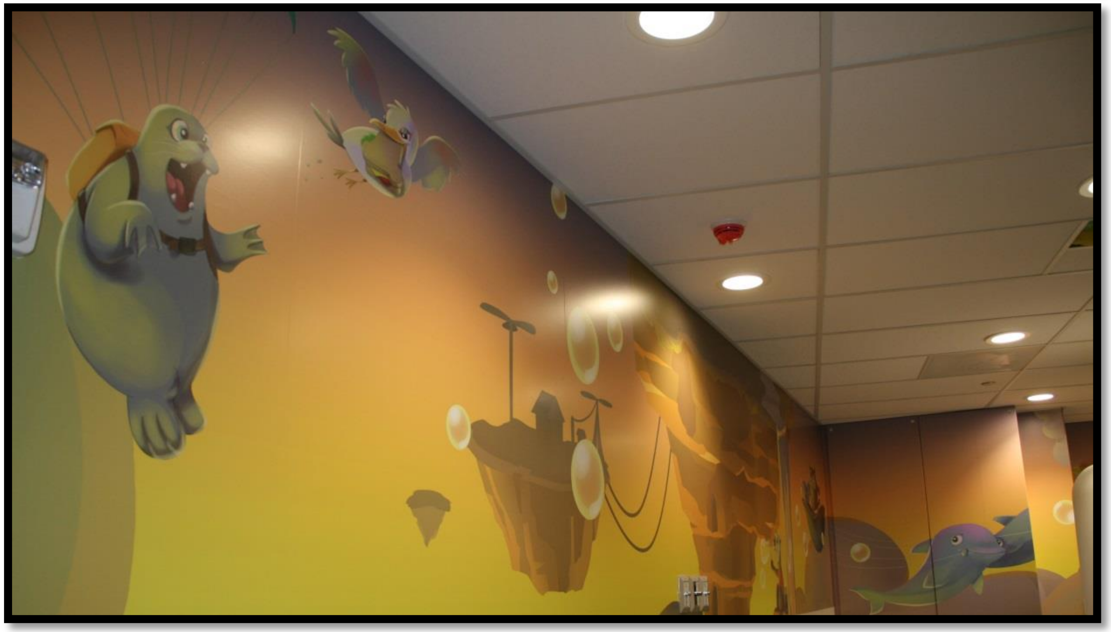

Ear protection

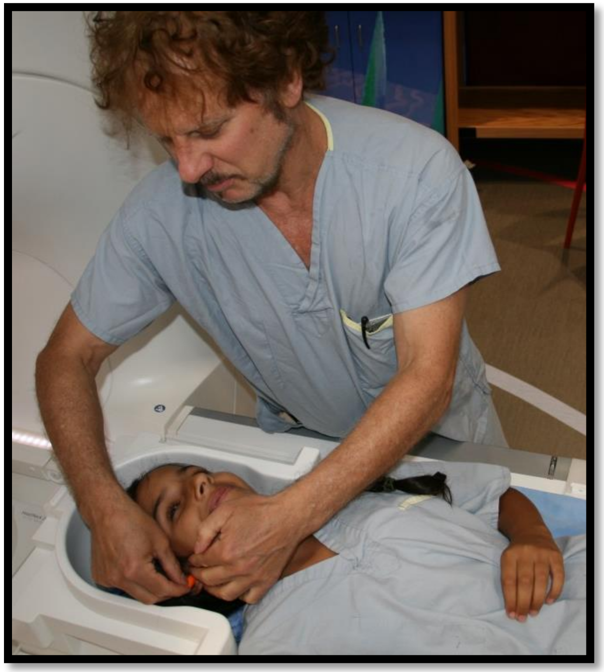

The MRI technologist will give you some earplugs and headphones to protect your ears from the loud noises.

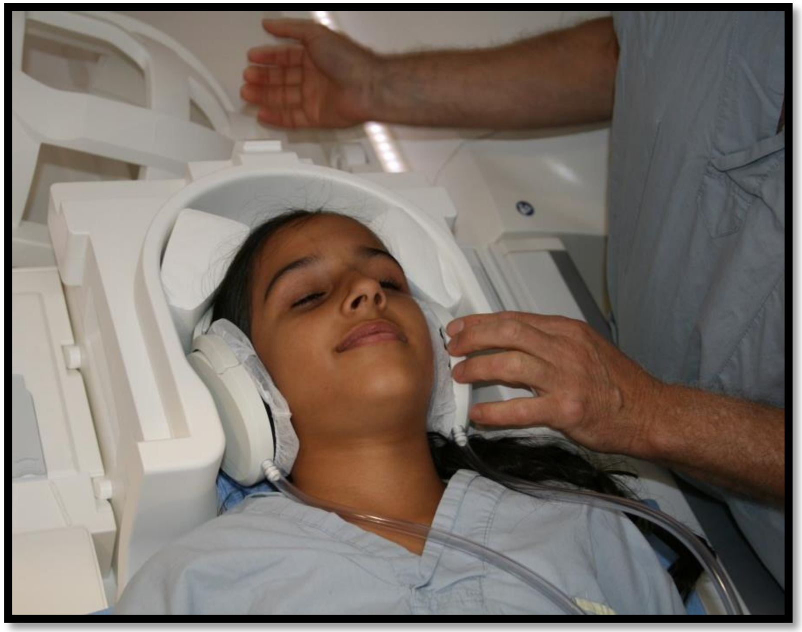

Emergency call bell

The technologist will give you an emergency call bell. You can squeeze this bell during your MRI if there is an emergency. If you think you might accidentally squeeze it, you can ask your caregiver to hold it for you.

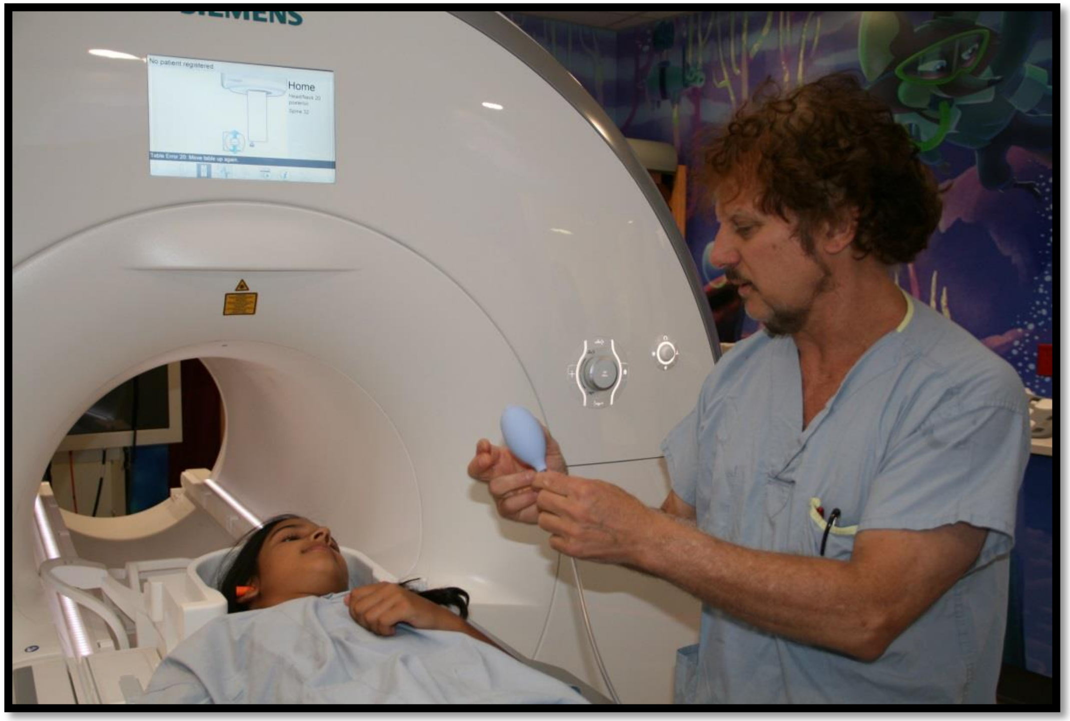

Getting ready for the scan: Coils

Coils are used to help take the pictures. The technologist will place a coil on your body where the pictures are being taken.

The head coil looks like an astronaut's helmet. For other coils please flip to the back of this book.

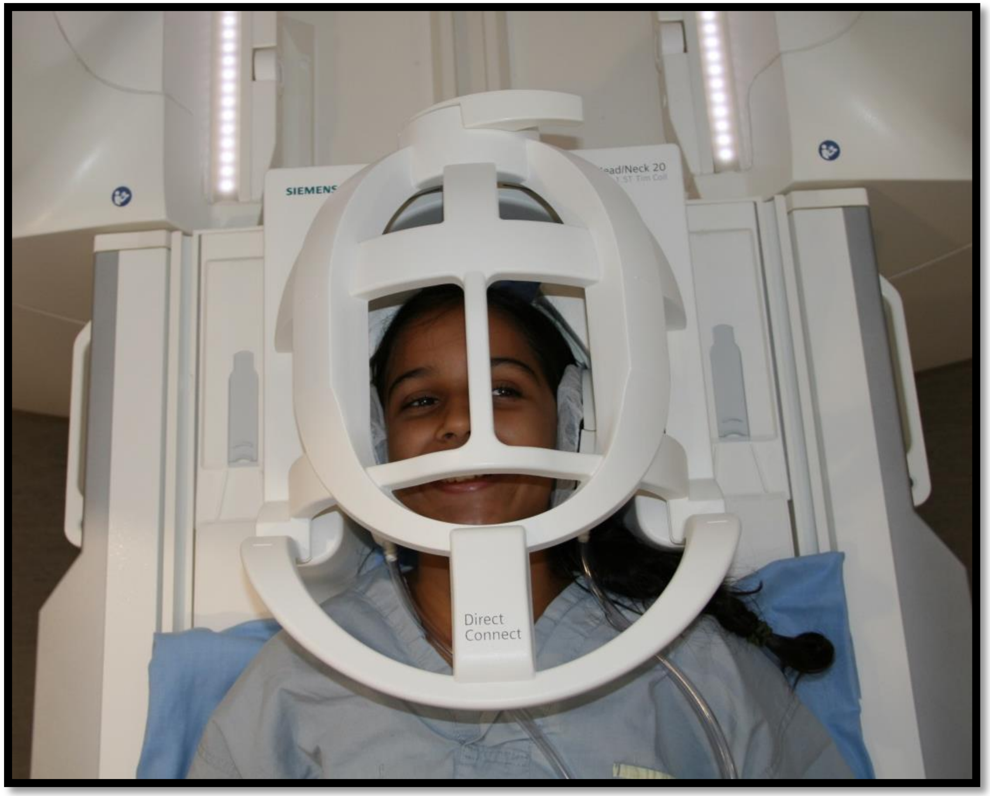

Getting ready for the scan: Seatbelts

The MRI technologist will buckle up a few seat belts. These seat belts keep you safe just like the ones in your car.

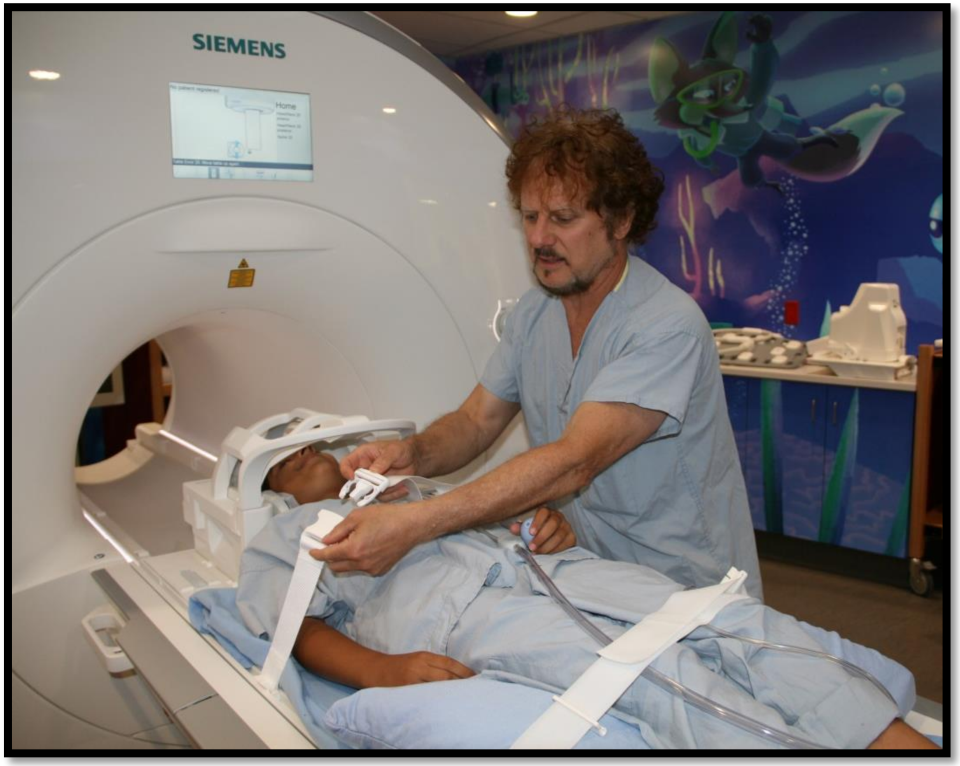

The red light

Before your pictures begin, the technologist will use a red light to help them centre your body for the pictures. You will not feel the red light.

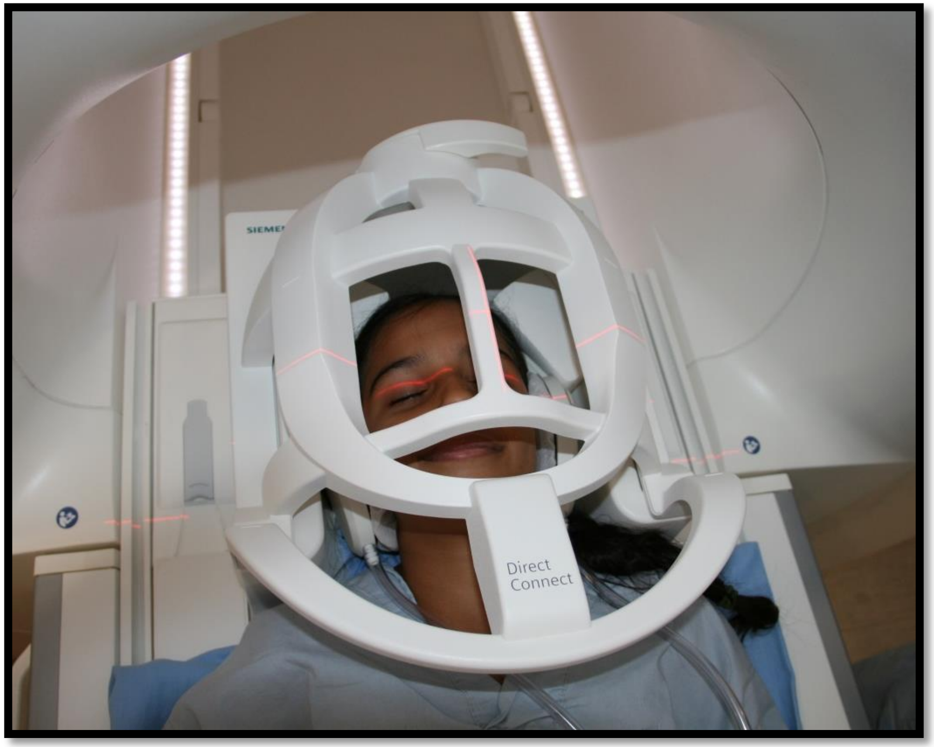

Someone can stay with you

An adult from your family can stay with you during your pictures. You may not see this person while you are in the tunnel.

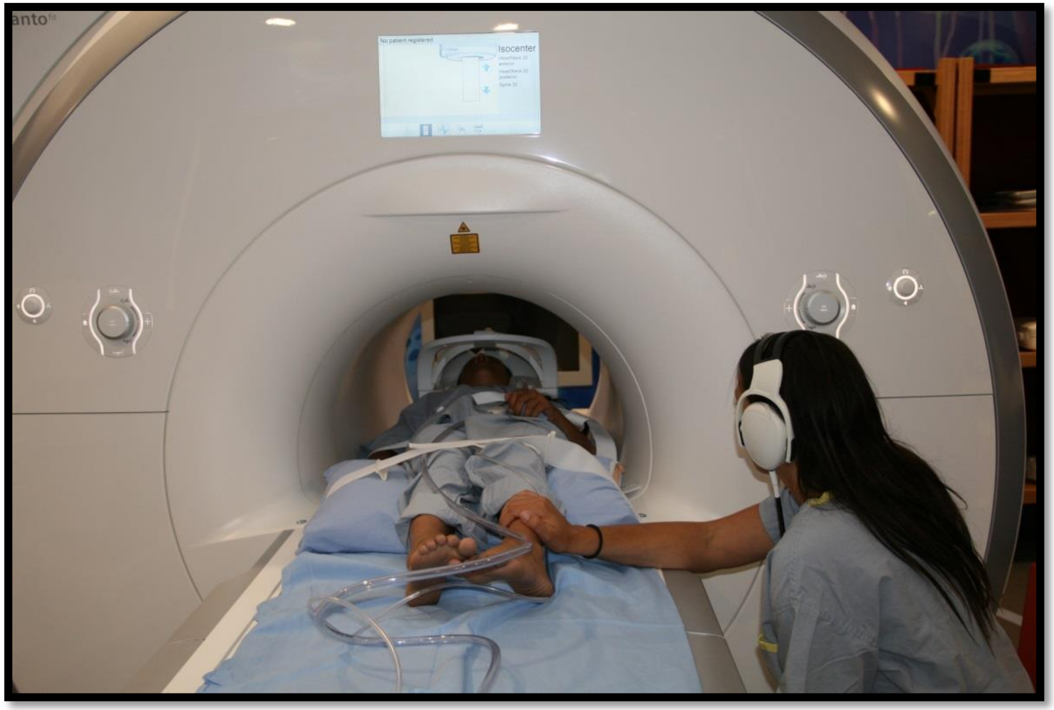

The control room

During your MRI, the technologist will go to the control room next door. They can see you through the window and talk to you over your headphones. If they ask you a question, answer them out loud.

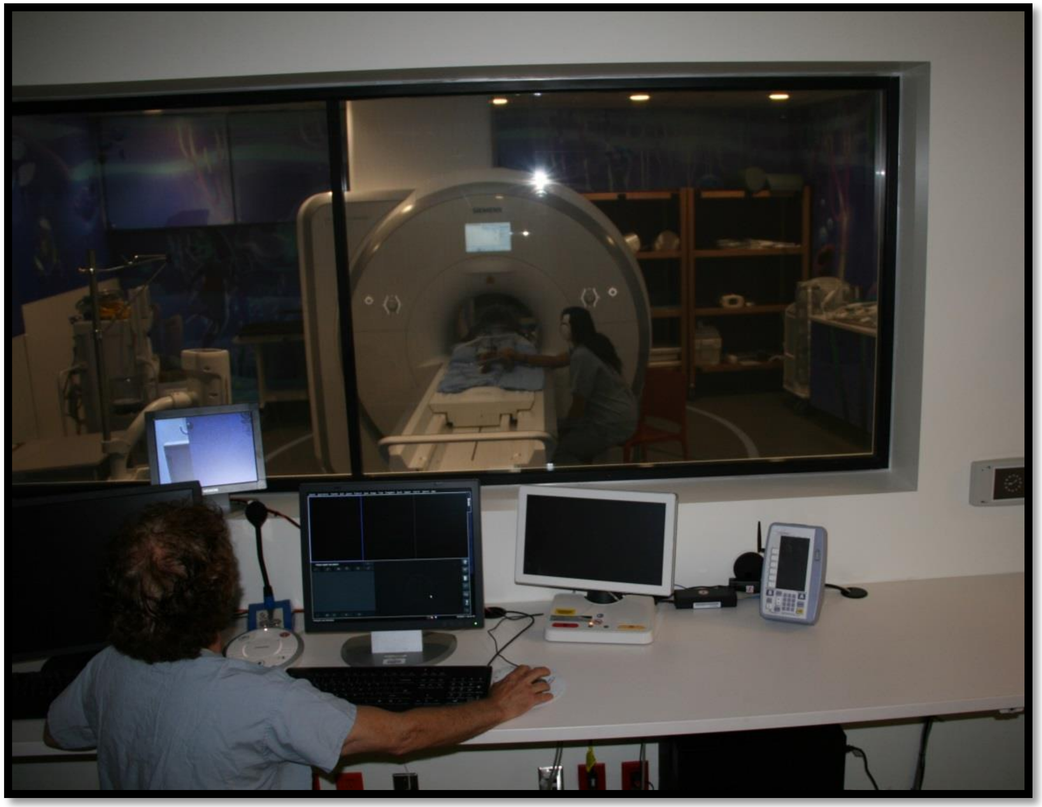

All finished

Once your MRI is finished, the technologist will bring you out of the MRI tunnel.

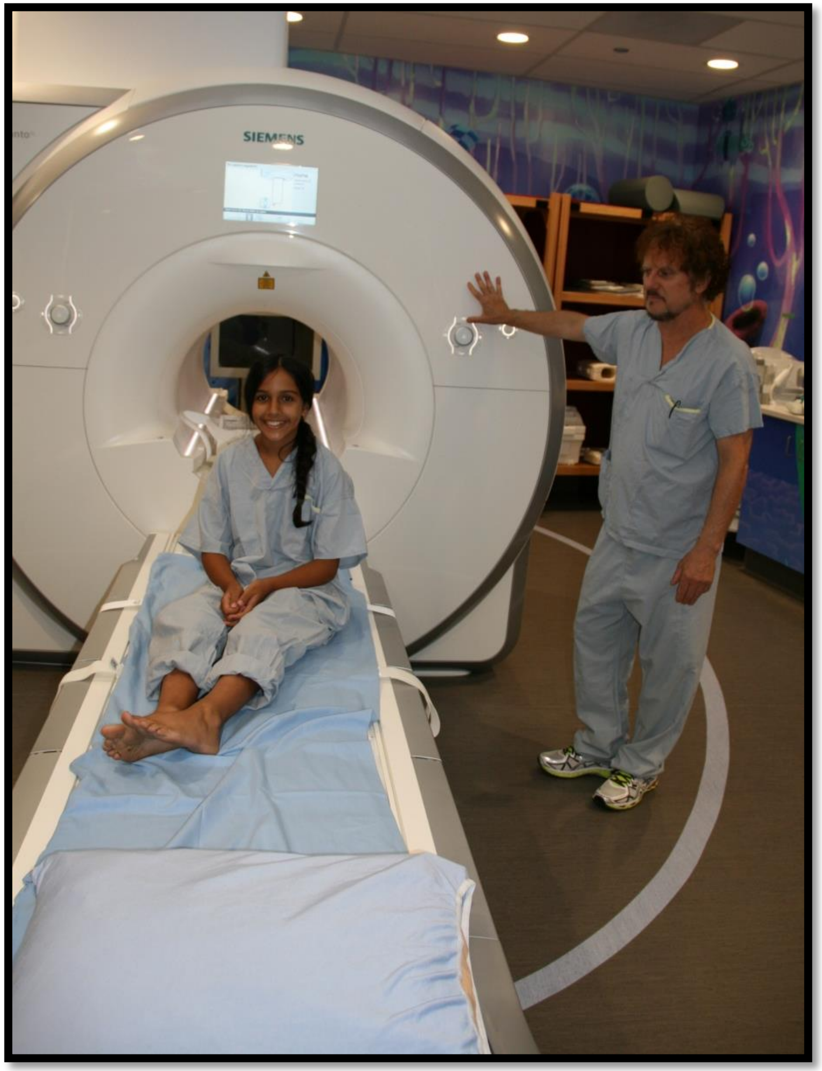

Other Coils

Coils

The MRI technologist will help you get comfortable before your scan begins. The technologist will place coils on you to help take the pictures. The type of coil looks different depending on what part of your body the MRI is taking pictures of.

Some coils come in two pieces that click together, others are one piece that are laid on top of you.

Knee Coils

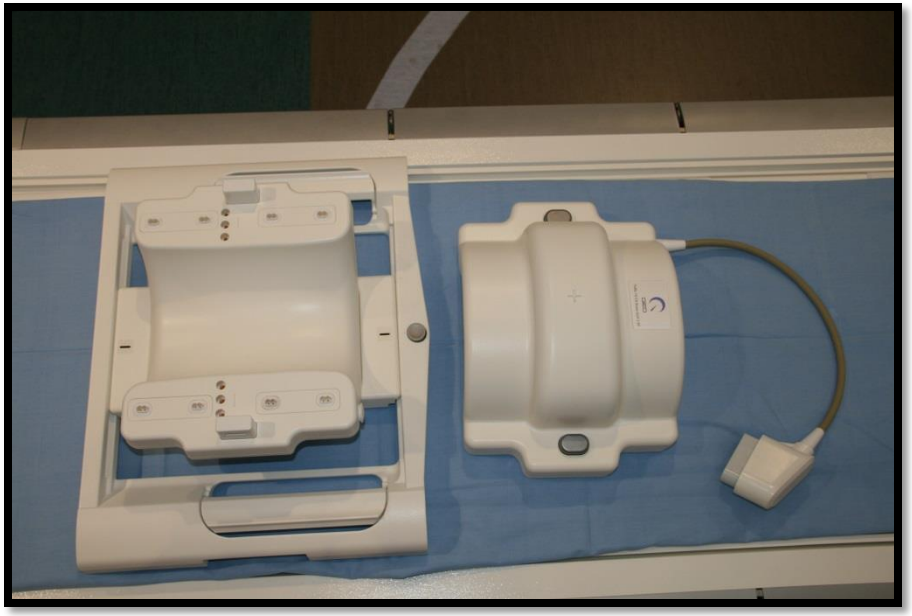

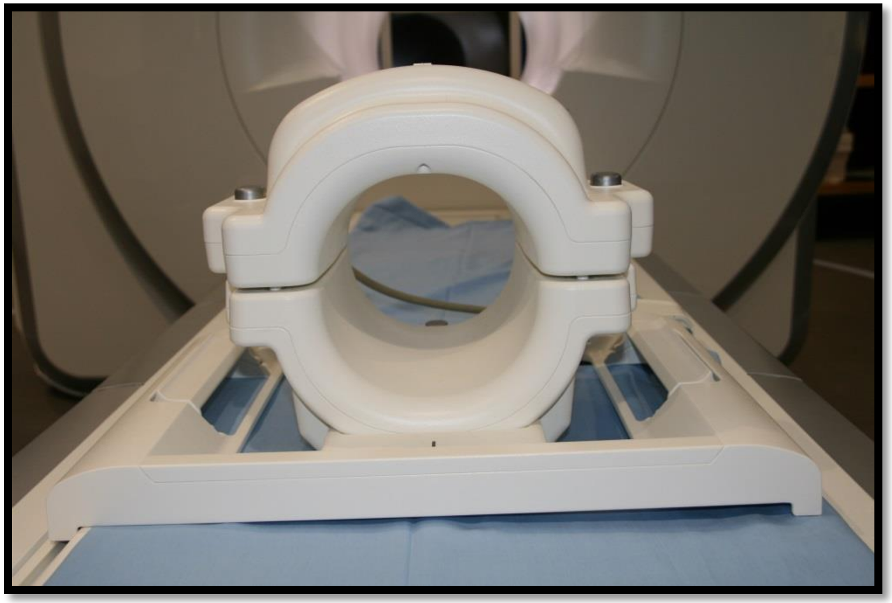

Knee Coils

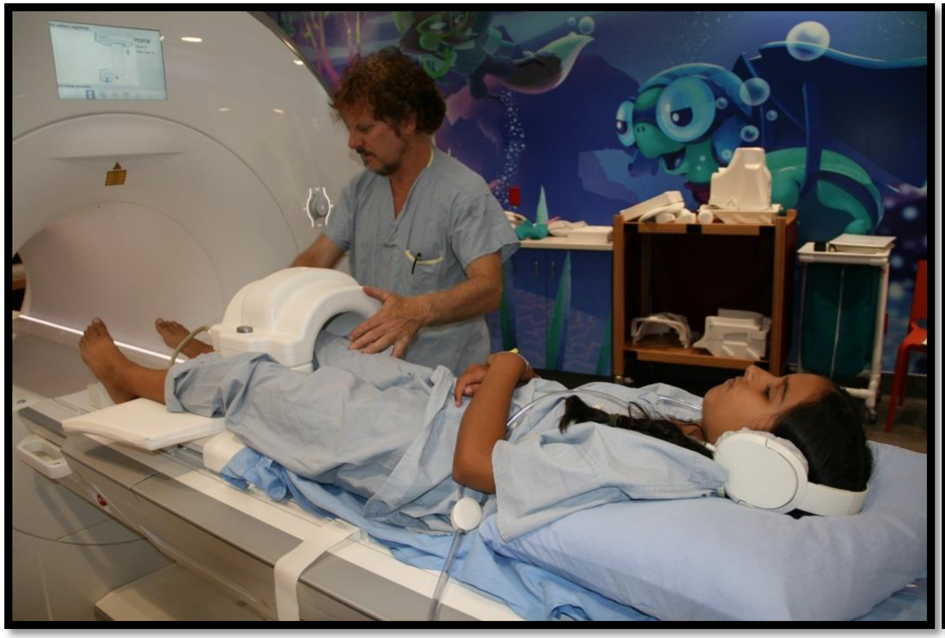

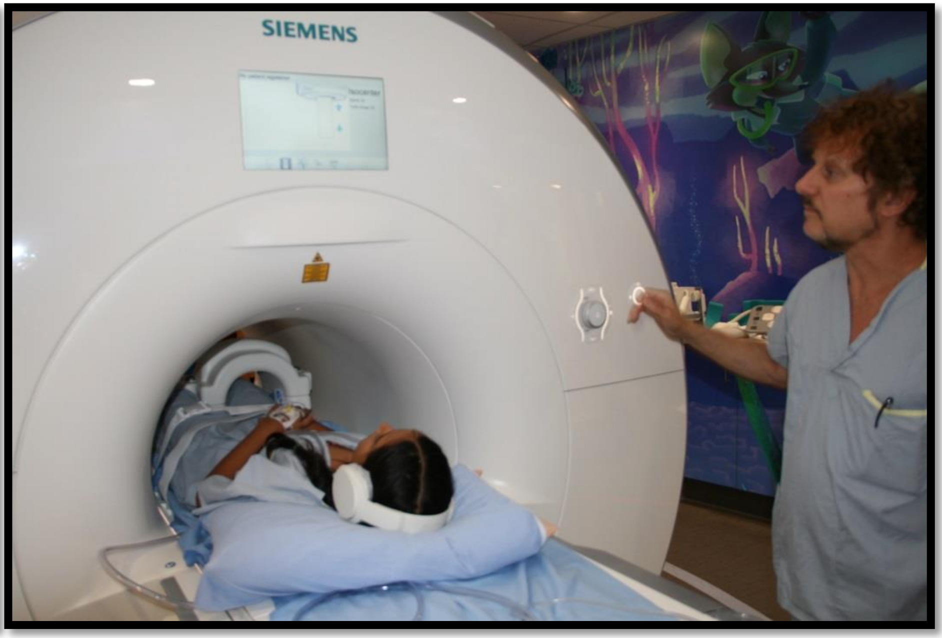

Hands Coils

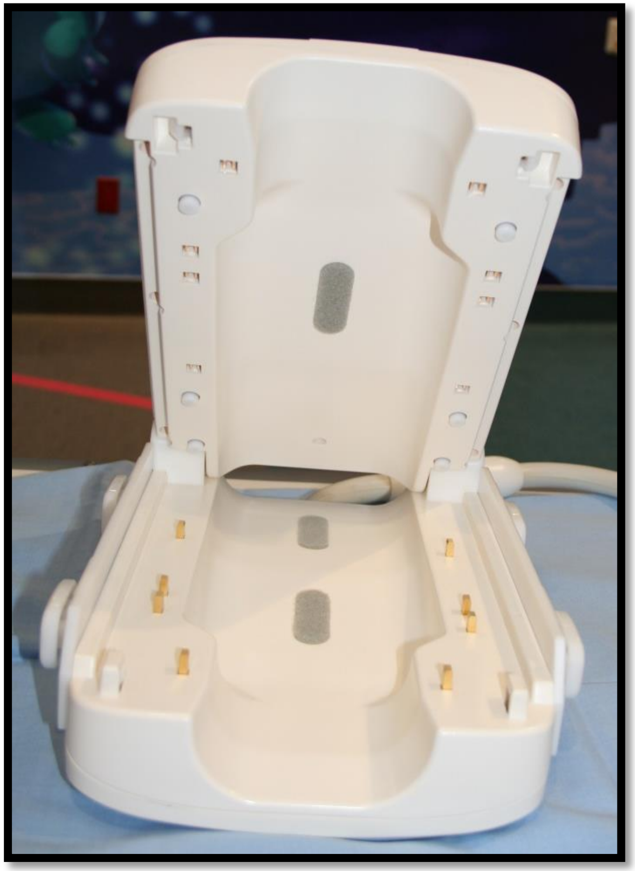

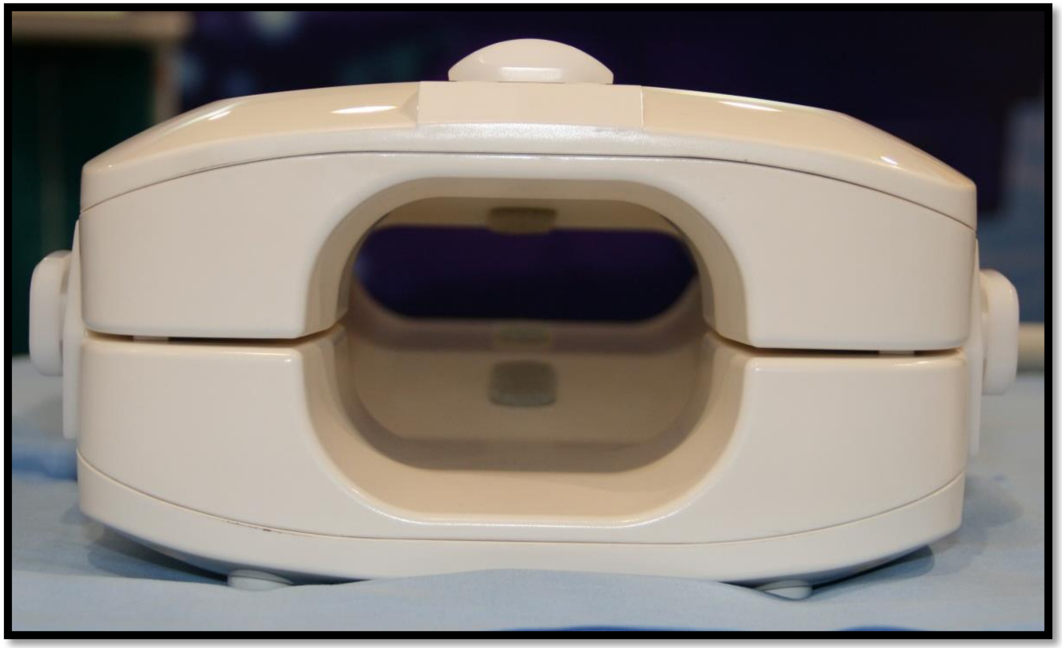

Hands Coils

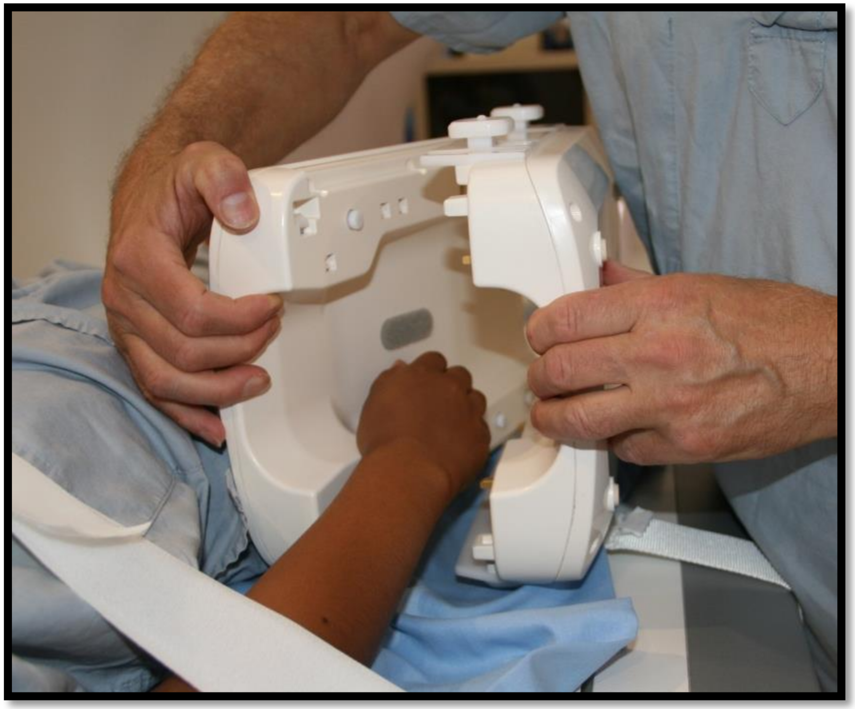

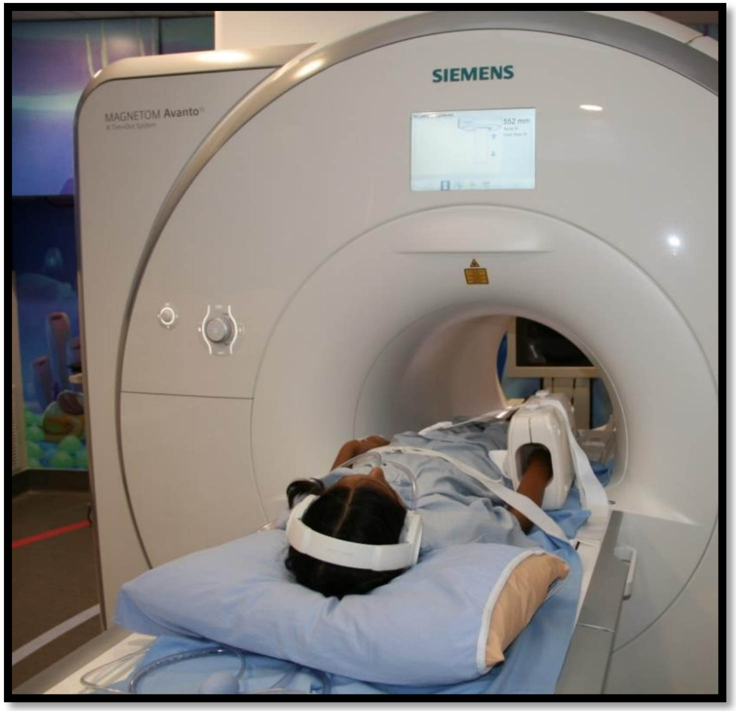

Foot Coils

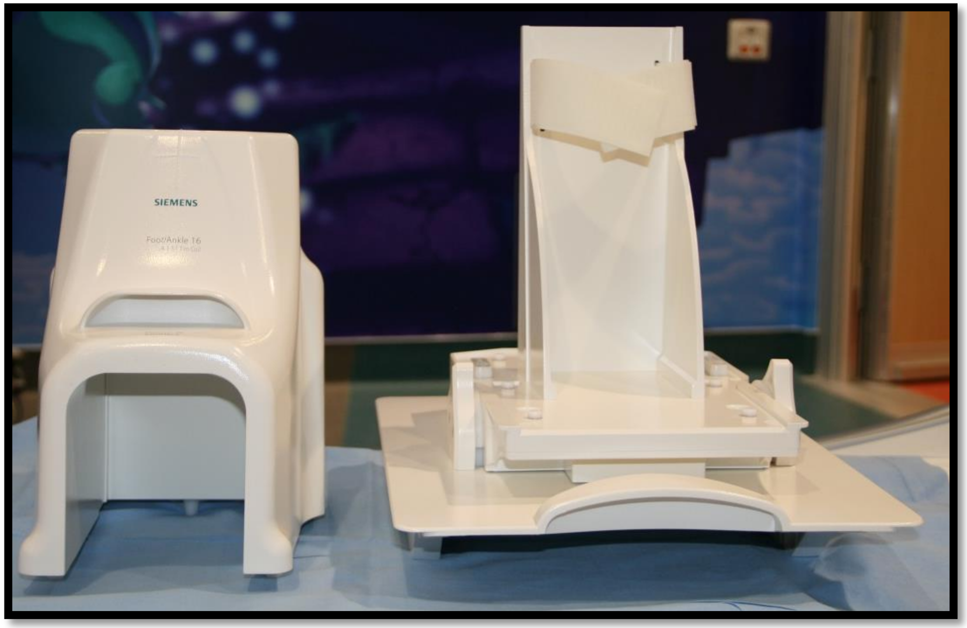

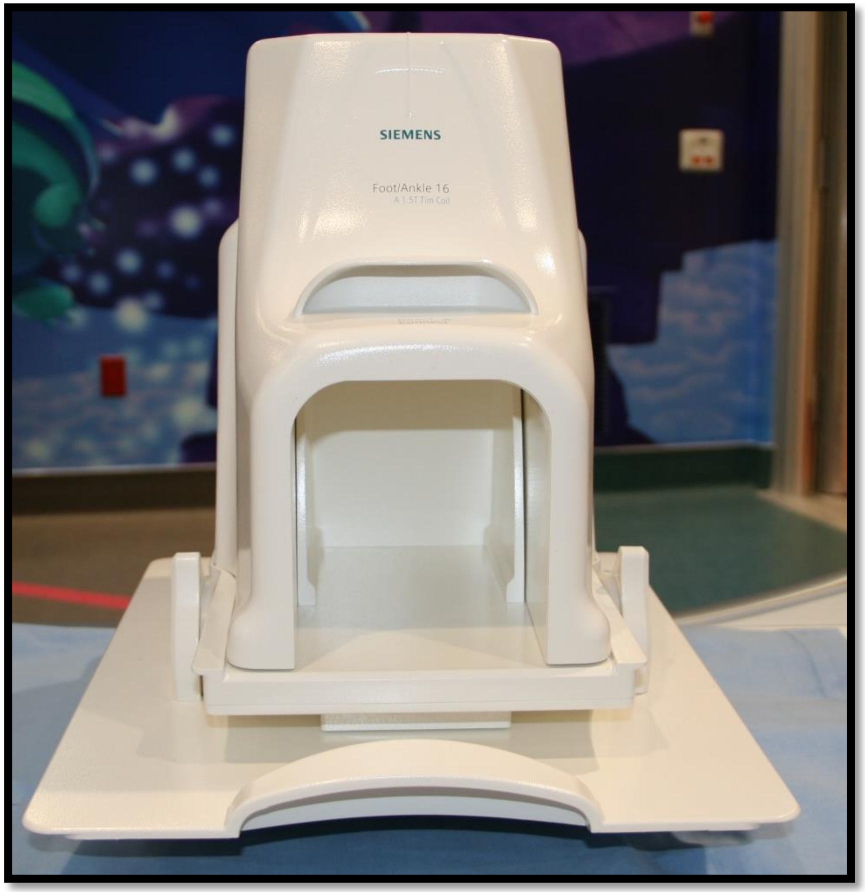

Foot Coils

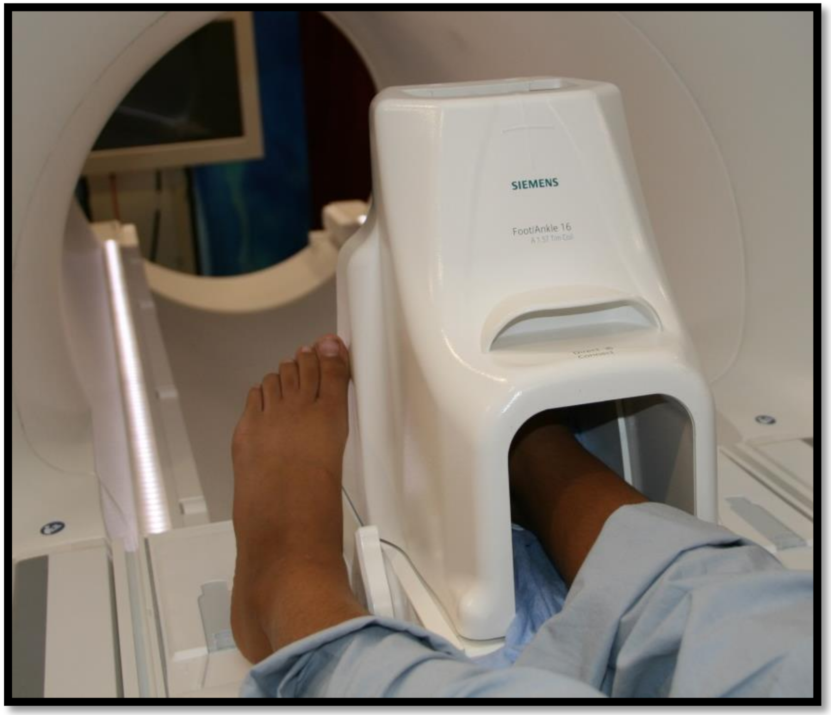

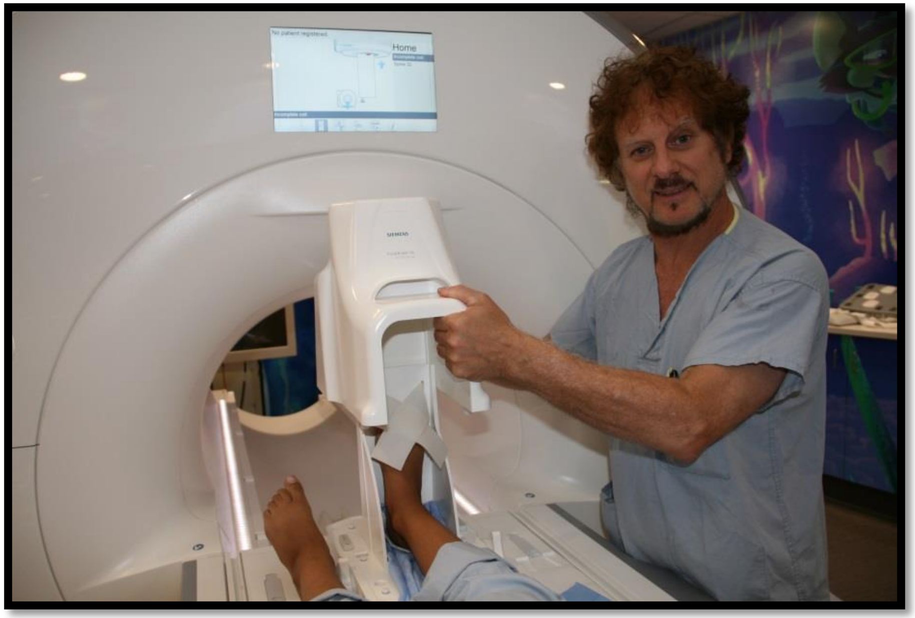

Leg Coils

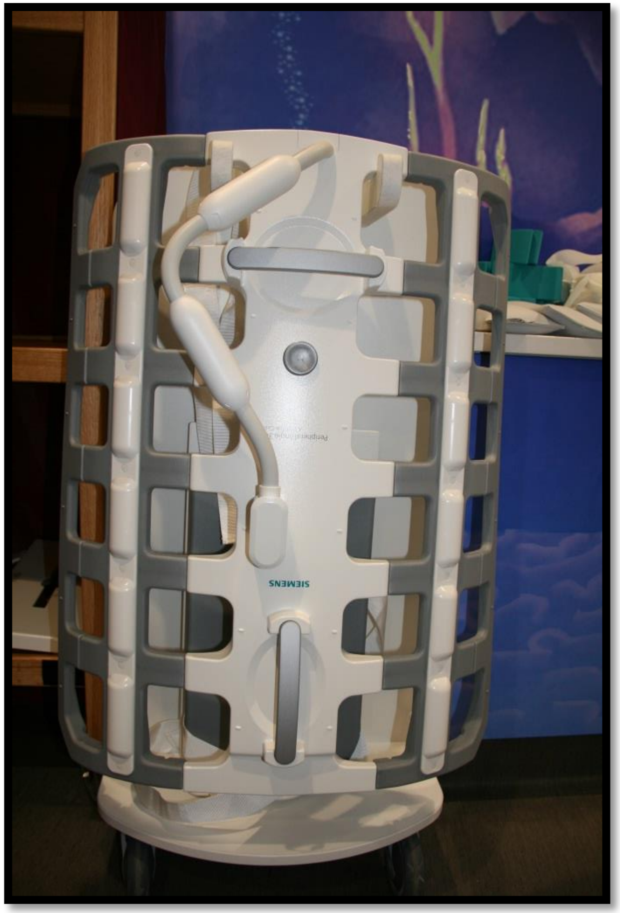

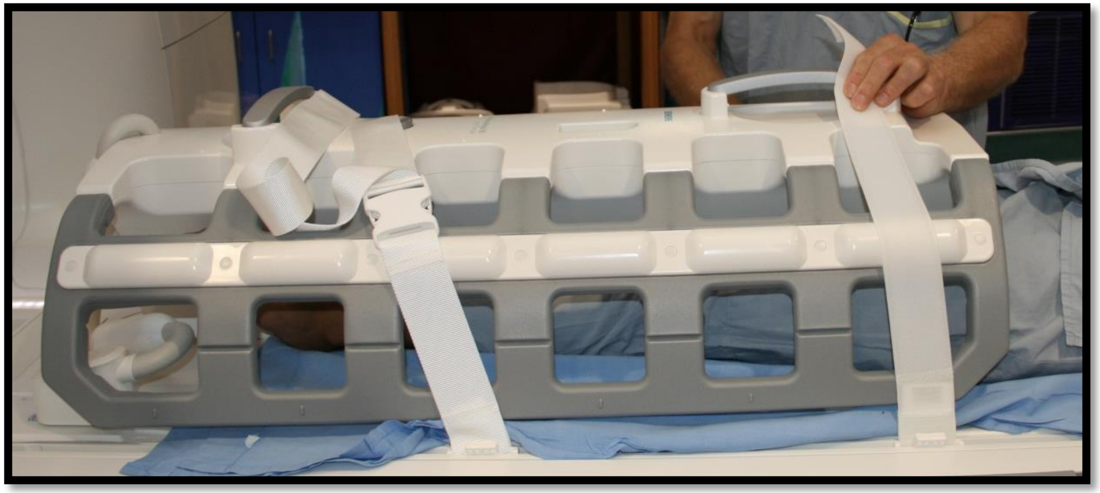

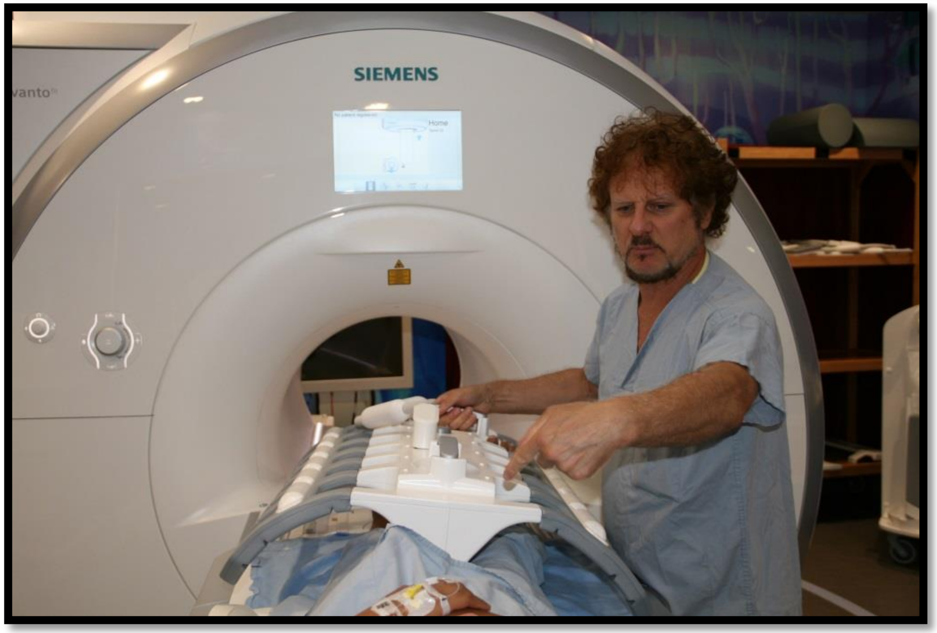

Body Coils

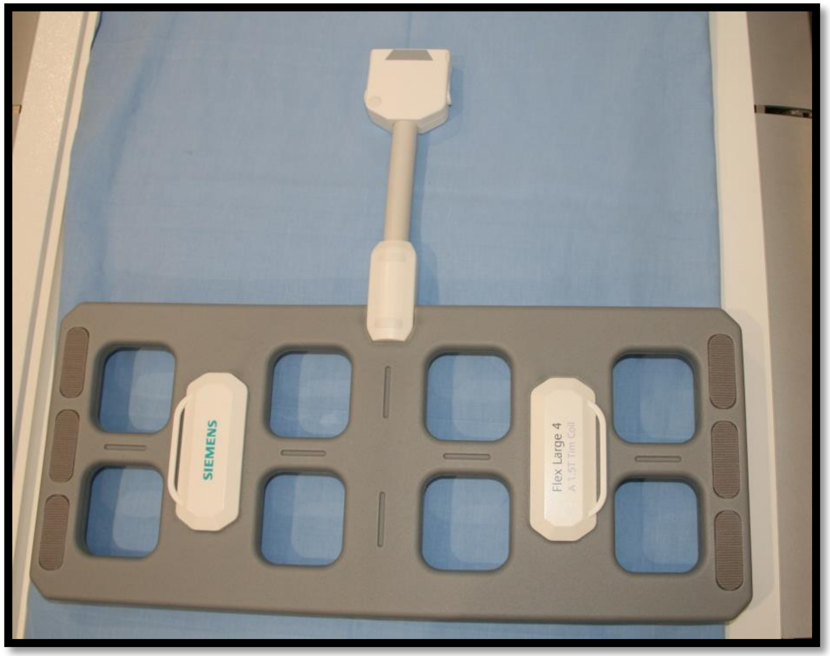

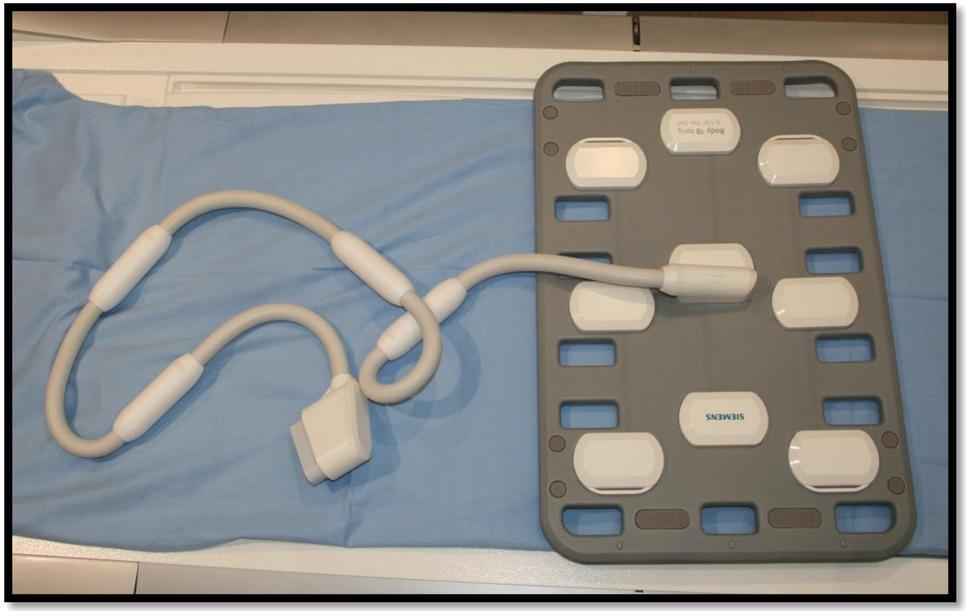

Body Coils

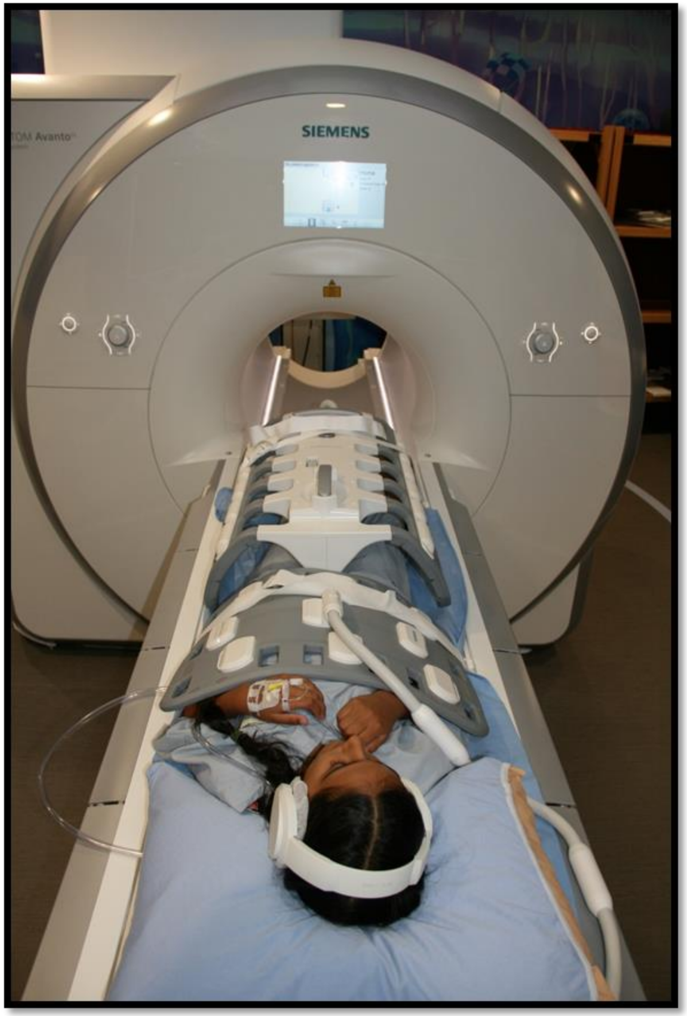
SIEMENS

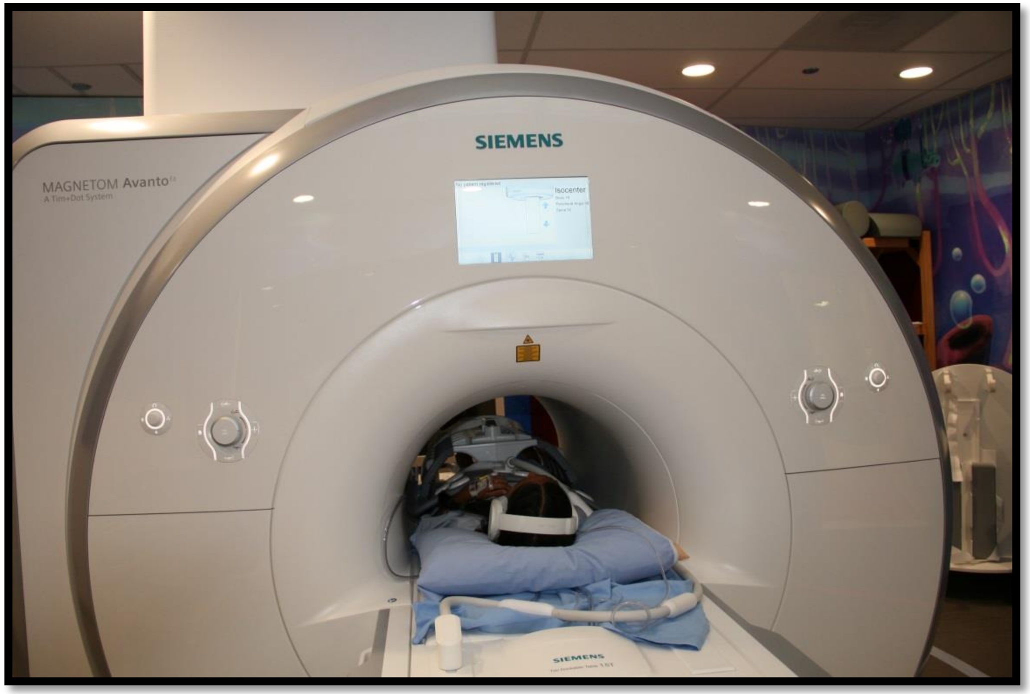
SIEMENS
MAGNETOM Avanto
A Tim+Dot System
Isocenter

Shoulder Coils

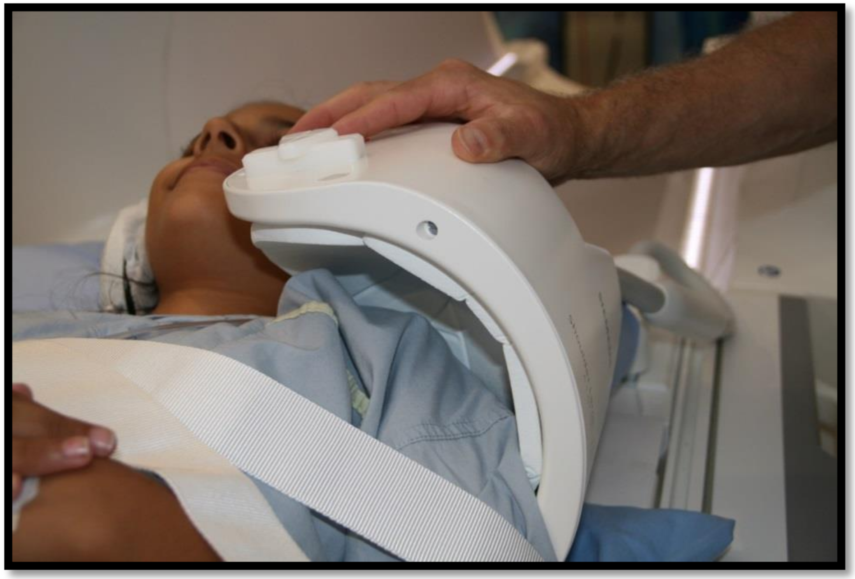

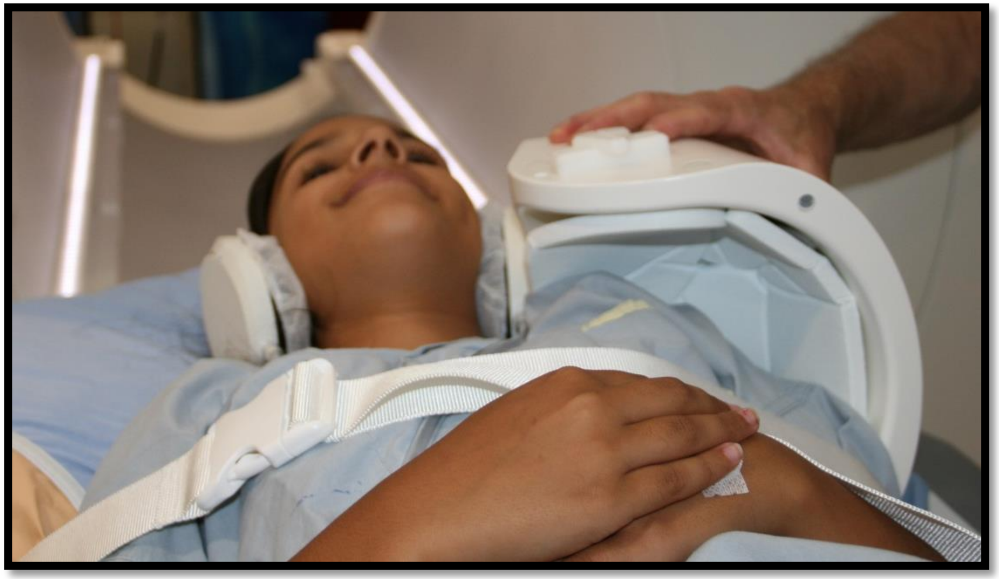

Contrast/GAD

Contrast/GAD

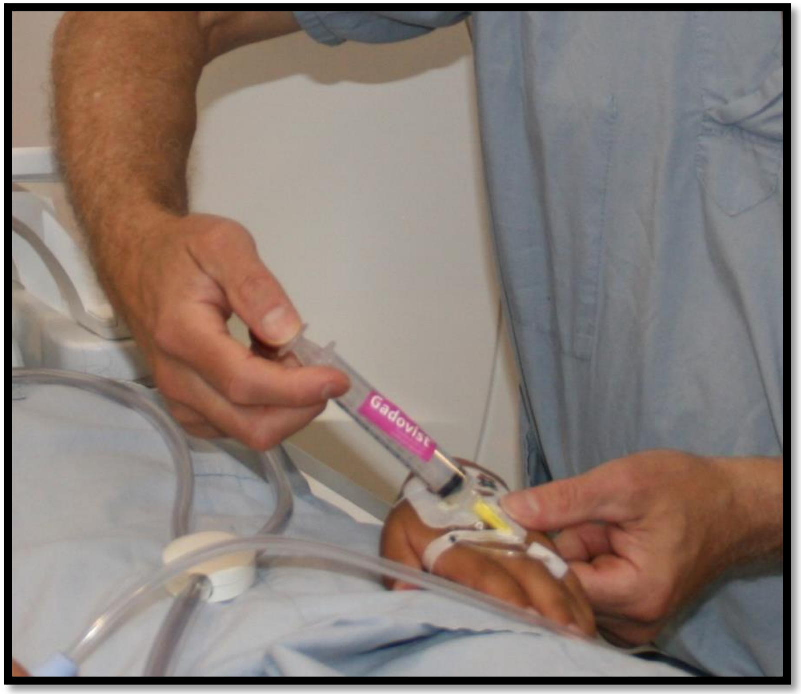

Some kids get a medicine called contrast or GAD that helps the pictures show up on the computer. This medicine goes into your body through an IV (intravenous).

An IV (intravenous) is a small straw that goes in your vein and used to give your body medicine.

Numbing Cream

The nurse will put some numbing cream on your hands and cover it with clear tape. This cream helps your skin from feeling the poke of the IV. Even though you have cream on both hands you will only get one IV.

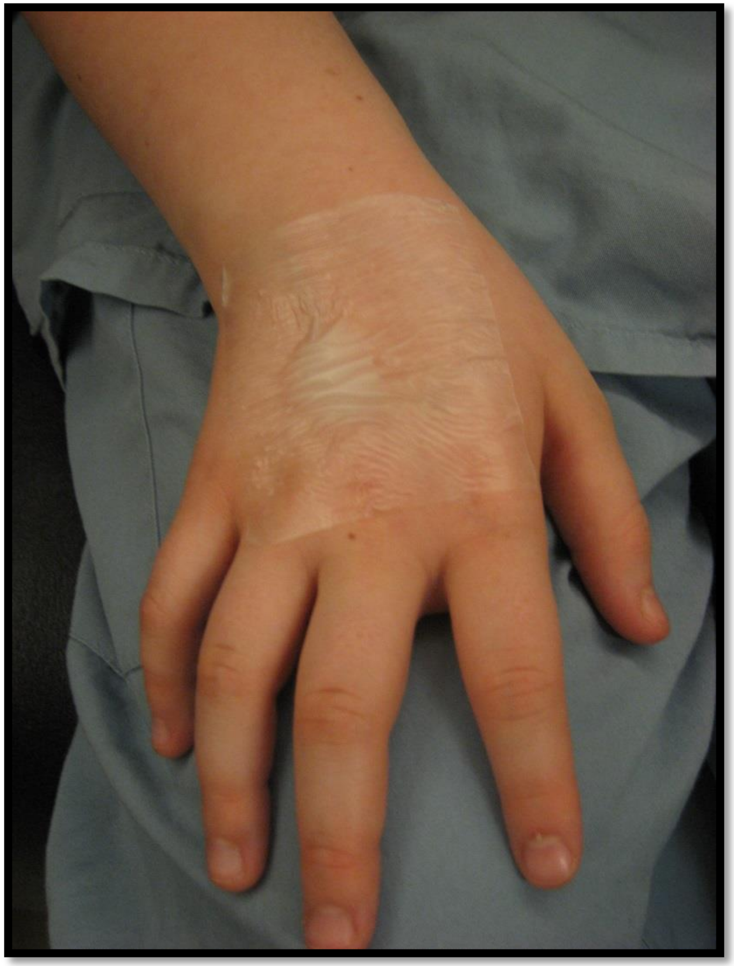

IV (intravenous)

Before you get your IV the nurse will peel off the clear plastic tape and wipe off the cream with a soft cloth and clean the area with a strong smelling wipe.

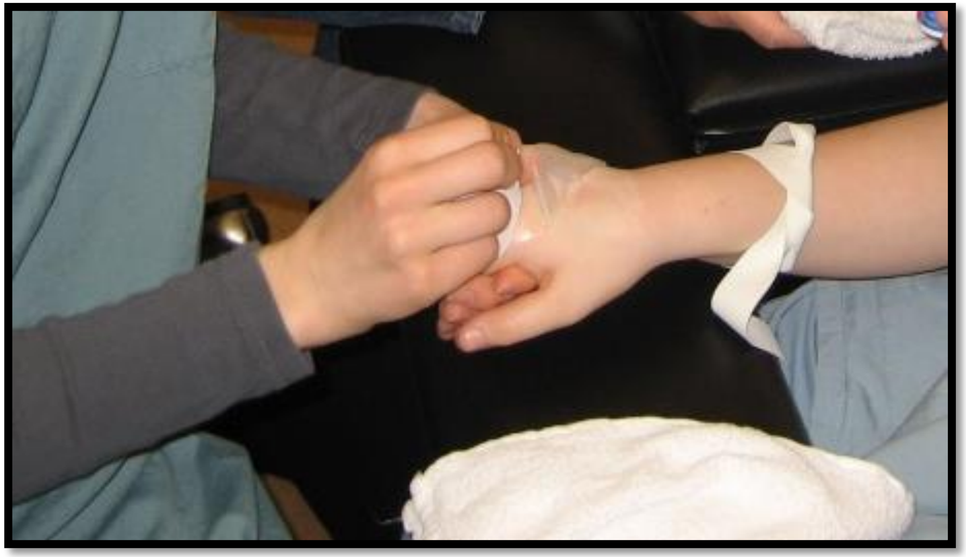

IV (intravenous)

The nurse will tie a stretchy elastic band around your arm before inserting the IV into your vein in your hand. Some kids like to blow bubbles as they prepare for their IV start.

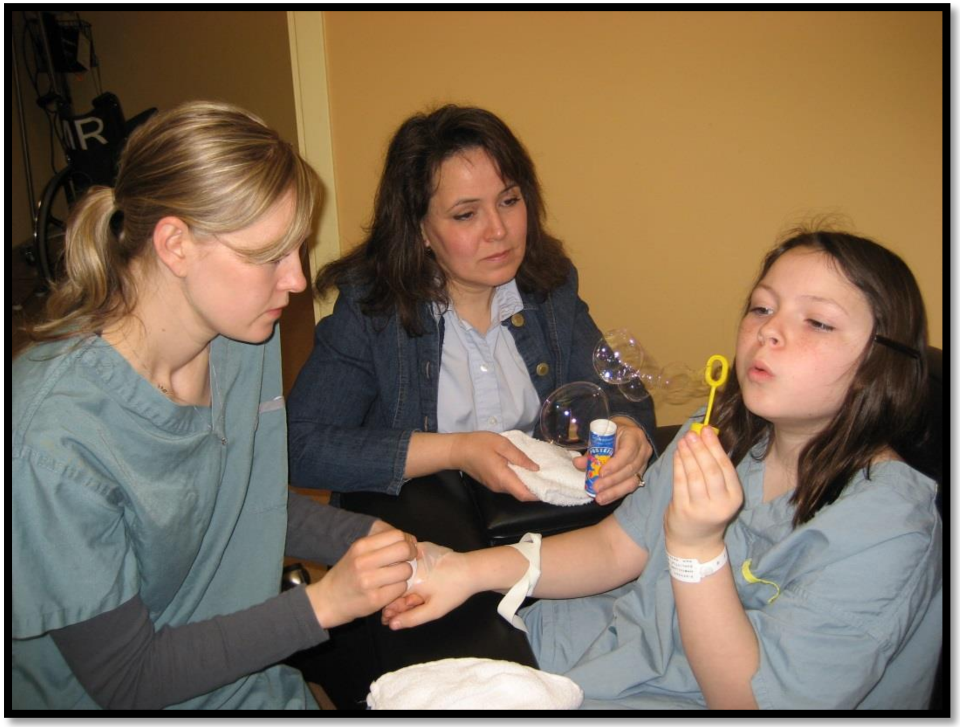

IV (intravenous)

This is what an IV looks like after it is inserted into your vein the nurse will secure it with some tape so it stays in place.

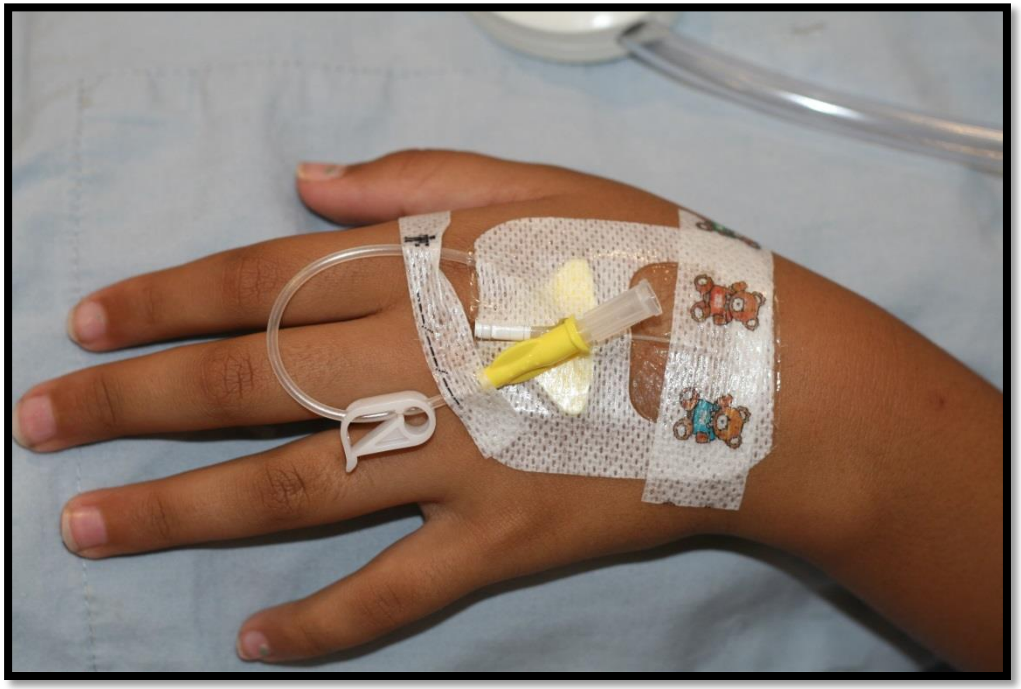

Supplement: Multimedia Appendix 1 [file jmir_v23i9e22942_app1.pdf]
