# Supplementary material for: Comparing a Virtual Reality–Based Simulation App (VR-MRI) With a Standard Preparatory Manual and Child Life Program for Improving Success and Reducing Anxiety During Pediatric Medical Imaging: Randomized Clinical Trial
Source: J Med Internet Res. 2021 Sep 22;23(9):e22942. doi: 10.2196/22942 (PMC8495586; doi:10.2196/22942)
Supplement: Multimedia Appendix 2 [file jmir_v23i9e22942_app2.pdf]

CONSORT-EHEALTH Checklist V1.6.2 Report	Manuscript Number	22942

(based on CONSORT-EHEALTH V1.6), available at [http://tinyurl.com/consort-ehealth-v1-6].

Date completed

11/23/2020 16:09:48

by

Chelsea Stunden

How does virtual reality compare to a standard preparatory manual and Child Life programming for improving success and reducing anxiety during paediatric medical imaging? A randomized clinical trial

TITLE

1a-i) Identify the mode of delivery in the title

"How does virtual reality compare to a standard preparatory manual and Child Life programming for improving success and reducing anxiety during paediatric medical imaging? A randomized clinical trial"

1a-ii) Non-web-based components or important co-interventions in title

"How does virtual reality compare to a standard preparatory manual and Child Life programming for improving success and reducing anxiety during paediatric medical imaging? A randomized clinical trial"

1a-iii) Primary condition or target group in the title

"How does virtual reality compare to a standard preparatory manual and Child Life programming for improving success and reducing anxiety during paediatric medical imaging? A randomized clinical trial"

ABSTRACT

1b-i) Key features/functionalities/components of the intervention and comparator in the METHODS section of the ABSTRACT

"At a 2-hour session, participants and their caregivers were instructed to prepare for a simulated MRI head scan using one of three randomly assigned preparation materials – the VR-MRI application, standard preparatory manual (SPM), or the hospital-based Child Life Preparatory Program (CLP). "

1b-ii) Level of human involvement in the METHODS section of the ABSTRACT

1b-iii) Open vs. closed, web-based (self-assessment) vs. face-to-face assessments in the METHODS section of the ABSTRACT

1b-iv) RESULTS section in abstract must contain use data

1b-v) CONCLUSIONS/DISCUSSION in abstract for negative trials

INTRODUCTION

2a-i) Problem and the type of system/solution

"the aim of this study was to compare the effectiveness of a custom-developed VR-based intervention to established hospital alternatives in preparing children aged 4-to-13 for a simulated medical imaging procedure. "

2a-ii) Scientific background, rationale: What is known about the (type of) system

Yes, page 2-3

Does your paper address CONSORT subitem 2b?

"Considering these points, the aim of this study was to compare the effectiveness of a custom-developed VR-based intervention to established hospital alternatives in preparing children aged 4-to-13 for a simulated medical imaging procedure. We hypothesized that a VR application (VR-MRI), based on experiential learning [37] and social cognitive theory [38], would be effective in preparing paediatric patients for a successful MRI experience and would reduce procedural anxieties. We secondarily hypothesized that:
-VR-MRI would reduce caregiver anxiety
-Children's anxiety would be related to their caregiver's anxiety
-More time practicing would result in a peri-procedural efficiency
-Caregivers would be satisfied with VR-MRI and it would be perceived as useful and easy to learn
-Children would be satisfied with VR-MRI and it would be perceived as fun to use"

METHODS

3a) CONSORT: Description of trial design (such as parallel, factorial) including allocation ratio

"A non-blinded, triple-arm randomized clinical trial was performed at a large provincial hospital in Vancouver, Canada between July 2019 and February 2020."

3b) CONSORT: Important changes to methods after trial commencement (such as eligibility criteria), with reasons

n/a

3b-i) Bug fixes, Downtimes, Content Changes

4a) CONSORT: Eligibility criteria for participants

"Participants were aged 4 – 13 years. Participants were excluded if they had mental disability, current concussion, significant visual and/or auditory impairment, inability to speak and understand English, history of seizures or epilepsy, facial or head wounds, or inability to move their head in all directions. All children provided assent and caregivers/legal guardians provided written consent. Participants received $20 and parking remuneration. "

4a-i) Computer / Internet literacy

4a-ii) Open vs. closed, web-based vs. face-to-face assessments:

Yes, this is described in section titled "outcome measurement"

4a-iii) Information giving during recruitment

4b) CONSORT: Settings and locations where the data were collected

"A non-blinded, triple-arm randomized clinical trial was performed at a large provincial hospital in Vancouver, Canada between July 2019 and February 2020. "

4b-i) Report if outcomes were (self-)assessed through online questionnaires

Outcomes that were self-assessed on a tablet included: caregiver anxiety (Short STAI), caregiver usability (USE questionnaire)

4b-ii) Report how institutional affiliations are displayed

5) CONSORT: Describe the interventions for each group with sufficient details to allow replication, including how and when they were actually administered

5-i) Mention names, credential, affiliations of the developers, sponsors, and owners

5-ii) Describe the history/development process

5-iii) Revisions and updating

5-iv) Quality assurance methods

5-v) Ensure replicability by publishing the source code, and/or providing screenshots/screen-capture video, and/or providing flowcharts of the algorithms used

5-vi) Digital preservation

5-vii) Access

"Participants were aged 4 – 13 years. Participants were excluded if they had mental disability, current concussion, significant visual and/or auditory impairment, inability to speak and understand English, history of seizures or epilepsy, facial or head wounds, or inability to move their head in all directions. All children provided assent and caregivers/legal guardians provided written consent. Participants received $20 and parking remuneration.
Participants were recruited through posters at the hospital, public libraries, community centres, and social media. We assigned participants 1:1:1 to VR-MRI or Child Life program (CLP) or standard preparatory manual (SPM) then tested for compliance during a simulated 6-minute MRI scan of head, designed to replicate an authentic scanning environment (Figure 11). Blinding was not feasible."

5-viii) Mode of delivery, features/functionalities/components of the intervention and comparator, and the theoretical framework

"The VR-MRI media was custom designed by the BC Children's Hospital Digital Lab. The development process was informed by the literature [39] and encompasses recent design strategy recommendations for VR [34]. Development of VR-MRI included iterative consultation and testing with a multidisciplinary team of approximately 8 stakeholders, including certified child life specialists, radiology technicians, child psychologists, health systems administrators, and the research team. The curriculum focuses on the same material included in the standard preparatory manual and Child Life programming: developing rapport with medical professionals, getting comfortable within the hospital setting and medical equipment, assessing reactions to pictures of a real MRI, discussing the upcoming medical procedure, getting comfortable with earplugs, headphones, loud noises, restraints, the head coil, going into and remaining inside the bore, and holding still.<br>We used an agile development methodology to cycle through user experience design, development, alpha testing, and beta testing [40]. Content in our study was custom developed in Unity and displayed on a Samsung S9 mobile phone that was embedded within a MERGE VR headset. The headset was selected for its balance of quality (eg, repeat use, compatibility with hygiene solutions, interpupillary adjustments, designed for children) and affordability, at $40 CAN. The headset requires a mobile phone to be inserted into an embedded front panel which is viewable to the users. We used AirServer Connect to mirror the VR-MRI sequence in real-time on a tablet device for caregivers to watch in parallel."		
**5-ix) Describe use parameters**		
**5-x) Clarify the level of human involvement**		
**5-xi) Report any prompts/reminders used**		
The details of the app prompts and reminders used are discussed in section titled "VR-MRI App"		
**5-xii) Describe any co-interventions (incl. training/support)**		
A research assistant helped to set up the VR hardware and monitored use.		
**6a) CONSORT: Completely defined pre-specified primary and secondary outcome measures, including how and when they were assessed**		
Yes, this is described in the section titled, "Outcome Measurement"		
**6a-i) Online questionnaires: describe if they were validated for online use and apply CHERRIES items to describe how the questionnaires were designed/deployed**		
**6a-ii) Describe whether and how "use" (including intensity of use/dosage) was defined/measured/monitored**		
**6a-iii) Describe whether, how, and when qualitative feedback from participants was obtained**		
**6b) CONSORT: Any changes to trial outcomes after the trial commenced, with reasons**		
"A non-blinded, triple-arm randomized clinical trial was performed at a large provincial hospital in Vancouver, Canada between July 2019 and February 2020. "		
**7a) CONSORT: How sample size was determined**		
**7a-i) Describe whether and how expected attrition was taken into account when calculating the sample size**		
**7b) CONSORT: When applicable, explanation of any interim analyses and stopping guidelines**		
Yes, this is described in the section titled, "Outcome Measurement"		
**8a) CONSORT: Method used to generate the random allocation sequence**		
"We assigned participants 1:1:1 to VR-MRI or Child Life program (CLP) or standard preparatory manual (SPM)"		
**8b) CONSORT: Type of randomisation; details of any restriction (such as blocking and block size)**		
Given the restricted availability of the simulator, child life specialists, and the<br>availability of participants, were unable to fully randomly allocate participants. As<br>such, participants were randomly assigned to the intervention options available<br>according to their individual availability. For example, if a participant is available on a Thursday that virtual reality and booklet are available, they were randomly allocated to one of these interventions.		
**9) CONSORT: Mechanism used to implement the random allocation sequence (such as sequentially numbered containers), describing any steps taken to conceal the sequence until interventions were assigned**		
1:1:1		
**10) CONSORT: Who generated the random allocation sequence, who enrolled participants, and who assigned participants to interventions**		
One research staff member was responsible for allocation and enrolling<br>participants (unblinded).		
**11a) CONSORT: Blinding - If done, who was blinded after assignment to interventions (for example, participants, care providers, those assessing outcomes) and how**		
**11a-i) Specify who was blinded, and who wasn't**		
Blinding was not feasible.		
**11a-ii) Discuss e.g., whether participants knew which intervention was the "intervention of interest" and which one was the "comparator"**		
**11b) CONSORT: If relevant, description of the similarity of interventions**		
The interventions are described in the methods section.		
**12a) CONSORT: Statistical methods used to compare groups for primary and secondary outcomes**		
"Statistical analysis was performed using the Statistical Package for the Social Sciences Version 22 (SPSS Inc, IL, USA). Continuous variables were expressed as mean (95% confidence interval) and ordinal variables with median (interquartile range). Categorical variables were expressed as percentage. Normality conditions were checked for all variables for applying proper test of significance. Many of the outcome variables were ordinal in nature and measured in score. Chi-square test was applied to test the independence of association between categorical variables. ANOVA (for normal distribution) or Kruskal-Wallis (for non-parametric distribution) was applied for one-way analysis to compare the three interventions' average scores among three time points. Post-hoc Bonferroni analysis was applied to statistically significant findings to confirm differences between groups. If equal variance assumption was not met during the ANOVA process, pairwise comparisons were based on the statistics of Dunnett's T3 [54]. To test for relationships between two continuous variables, we used bivariate Pearson Correlation. In the case of missing values, a single value was filled for each missing value by averaging the collected scores for that participant."		
**12a-i) Imputation techniques to deal with attrition / missing values**		
"In the case of missing values, a single value was filled for each missing value by averaging the collected scores for that participant."		
**12b) CONSORT: Methods for additional analyses, such as subgroup analyses and adjusted analyses**		
none		
RESULTS		
**13a) CONSORT: For each group, the numbers of participants who were randomly assigned, received intended treatment, and were analysed for the primary outcome**		
"A total of 92 participants were recruited over the study period; one did not consent and one participant did not show to the appointment. One participant provided consent initially, but was later withdrawn. As such, a total of 89 participants were enrolled. Of the consenting participants, five were excluded due to equipment malfunction. This left 84 participants in the analysis (VR-MRI n=30, SPM n=24, CLP n=30). "		
**13b) CONSORT: For each group, losses and exclusions after randomisation, together with reasons**		
Yes, Figure 10.		
**13b-i) Attrition diagram**		
**14a) CONSORT: Dates defining the periods of recruitment and follow-up**		
" between July 2019 and February 2020. "		
**14a-i) Indicate if critical "secular events" fell into the study period**		
**14b) CONSORT: Why the trial ended or was stopped (early)**		
n/a		
**15) CONSORT: A table showing baseline demographic and clinical characteristics for each group**		
yes, see section "demographic and clinical characteristics"		
**15-i) Report demographics associated with digital divide issues**		
"Demographic and clinical characteristics of patients are shown in Table 1. Most participants were male (60.7% total) and had no history of MRI (91.7% total) or simulator experience (96.4% total). About half of the participants had experience with other medical imaging procedures (48.8% total) and many had used virtual reality prior to participating in the study (79.5% total). Chi-square tests were conducted on demographic variables and ANOVA on continuous variables. No significant differences in any of the demographic variables were found amongst the groups (p > 0.05)."		
**16a) CONSORT: For each group, number of participants (denominator) included in each analysis and whether the analysis was by original assigned groups**		
**16-i) Report multiple "denominators" and provide definitions**		
yes, this is included throughout our results section.		

**16-ii) Primary analysis should be intent-to-treat**		
**17a) CONSORT: For each primary and secondary outcome, results for each group, and the estimated effect size and its precision (such as 95% confidence interval)**		
yes, we have included this		
**17a-i) Presentation of process outcomes such as metrics of use and intensity of use**		
**17b) CONSORT: For binary outcomes, presentation of both absolute and relative effect sizes is recommended**		
yes, we have included this		
**18) CONSORT: Results of any other analyses performed, including subgroup analyses and adjusted analyses, distinguishing pre-specified from exploratory**		
n/a		
**18-i) Subgroup analysis of comparing only users**		
**19) CONSORT: All important harms or unintended effects in each group**		
"One child (5 years old) indicated eye strain and a blurry image when viewing VR-MRI that could not be mitigated by interpupillary adjustments. Two children (ages 6 and 4 years) reported the dinosaur graphic in VR-MRI was "scary". Six participants (ages 4 to 12) in the manual groups expressed being scared of pictures in the manual; particularly the sections of the intravenous or coil pictures. No other side effects were reported. "		
**19-i) Include privacy breaches, technical problems**		
**19-ii) Include qualitative feedback from participants or observations from staff/researchers**		
DISCUSSION		
**20) CONSORT: Trial limitations, addressing sources of potential bias, imprecision, multiplicity of analyses**		
**20-i) Typical limitations in ehealth trials**		
"There are several potential limitations to this study.<br>Our methodology focused on self-reporting of anxiety for children and parents. We used the Short STAI for caregivers, but the VPT for children which may introduce confounding. The results may also have been affected if users did not fully understand the meaning or how to complete the surveys after instructions. There may have been response bias, as children often consciously or subconsciously give responses that they think adults want to hear.<br>Our study is also subject to several additional biases. The study was subject to information and selection biases, as we recruited participants though posters at the hospital, public libraries, and social media, and provided remuneration and parking reimbursement. Motivation and reported outcomes related to using the materials could have been impacted by these extrinsic motivations (eg, remuneration). The study was also unblinded to participants and research staff due to practicalities of the preparatory processes and logistical limitations.<br>Our study has a small sample size that just met the requirements of our power calculation. The effect sizes were smaller than we anticipated between groups and, as such, this study is at risk for type II error (accepting a null hypothesis that is actually false). Five participants did not have metrics for movement because of technology malfunction. In our study, we used the MERGE VR headset because no other HMDs have been indicated for use specifically with children. This headset is indicated to match interpupillary distance of children 10 years and older. Younger children may have smaller interpupillary distance than what can be adjusted for. One study participant reported eye strain and a blurry image (age 5) that could not be mitigated by adjustment, which is likely a result of that limitation. Currently available consumer-grade virtual reality hardware has not been designed for use with younger children and, in some cases, might not be adjustable for the parameters required by them."		
**21) CONSORT: Generalisability (external validity, applicability) of the trial findings**		
**21-i) Generalizability to other populations**		
**21-ii) Discuss if there were elements in the RCT that would be different in a routine application setting**		
**22) CONSORT: Interpretation consistent with results, balancing benefits and harms, and considering other relevant evidence**		
**22-i) Restate study questions and summarize the answers suggested by the data, starting with primary outcomes and process outcomes (use)**		
Yes, we have reported these findings in the discussion section.		
**22-ii) Highlight unanswered new questions, suggest future research**		
Other information		
**23) CONSORT: Registration number and name of trial registry**		
U.S. National Library of Medicine (#NCT03931382)		
**24) CONSORT: Where the full trial protocol can be accessed, if available**		
none		
**25) CONSORT: Sources of funding and other support (such as supply of drugs), role of funders**		
none		
**X26-i) Comment on ethics committee approval**		
**x26-ii) Outline informed consent procedures**		
**X26-iii) Safety and security procedures**		
**X27-i) State the relation of the study team towards the system being evaluated**		
